# High frequency of an otherwise rare phenotype in a small and isolated tiger population

**DOI:** 10.1073/pnas.2025273118

**Published:** 2021-09-13

**Authors:** Vinay Sagar, Christopher B. Kaelin, Meghana Natesh, P. Anuradha Reddy, Rajesh K. Mohapatra, Himanshu Chhattani, Prachi Thatte, Srinivas Vaidyanathan, Suvankar Biswas, Supriya Bhatt, Shashi Paul, Yadavendradev V. Jhala, Mayank M. Verma, Bivash Pandav, Samrat Mondol, Gregory S. Barsh, Debabrata Swain, Uma Ramakrishnan

**Affiliations:** a National Centre for Biological Sciences, Tata Institute of Fundamental Research, Bangalore 560065, India; b Department of Genetics, Stanford University, Palo Alto, CA 94309; c HudsonAlpha Institute for Biotechnology, Huntsville, AL 35806; d Biology Department, Indian Institute of Science Education and Research, Tirupati 411008, India; e Laboratory for Conservation of Endangered Species, Center for Cellular & Molecular Biology, Hyderabad 500048, India; f Nandankanan Biological Park, Bhubaneswar 754005, India; g World Wide Fund for Nature - India, New Delhi 110003 India; h Foundation for Ecological Research, Advocacy and Learning, Auroville Post, Tamil Nadu 605101 India; i Wildlife Institute of India, Dehradun 248001, India; j Odisha Forest Department, Bhubaneswar 751023, India; k National Tiger Conservation Authority, Wildlife Institute of India Tiger Cell, Wildlife Institute of India, Dehradun 248001, India; l State Forest Research Institute, Jabalpur 482008, India; m Former Member Secretary, National Tiger Conservation Authority, New Delhi 110003, India; n Former Principal Chief Conservator of Forest and Head of Forest Force, Indian Forest Service, Bhubaneswar 751023, India; o DBT - Wellcome Trust India Alliance, Hyderabad 500034, India

**Keywords:** pseudomelanism, drift, selection, inbreeding, genetics

## Abstract

Most endangered species exist today in small populations, many of which are isolated. Evolution in such populations is largely governed by genetic drift. Empirical evidence for drift affecting striking phenotypes based on substantial genetic data are rare. Approximately 37% of tigers (*Panthera tigris*) in the Similipal Tiger Reserve (in eastern India) are pseudomelanistic, characterized by wide, merged stripes. Camera trap data across the tiger range revealed the presence of pseudomelanistic tigers only in Similipal. We investigated the genetic basis for pseudomelanism and examined the role of drift in driving this phenotype’s frequency. Whole-genome data and pedigree-based association analyses from captive tigers revealed that pseudomelanism cosegregates with a conserved and functionally important coding alteration in *Transmembrane Aminopeptidase Q* (*Taqpep*), a gene responsible for similar traits in other felid species. Noninvasive sampling of tigers revealed a high frequency of the Taqpep p.H454Y mutation in Similipal (12 individuals, allele frequency = 0.58) and absence from all other tiger populations (395 individuals). Population genetic analyses confirmed few (minimal number) tigers in Similipal, and its genetic isolation, with poor geneflow. Pairwise F_ST_ (0.33) at the mutation site was high but not an outlier. Similipal tigers had low diversity at 81 single nucleotide polymorphisms (mean heterozygosity = 0.28, SD = 0.27). Simulations were consistent with founding events and drift as possible drivers for the observed stark difference of allele frequency. Our results highlight the role of stochastic processes in the evolution of rare phenotypes. We highlight an unusual evolutionary trajectory in a small and isolated population of an endangered species.

Several recent studies demonstrate that biodiversity is declining globally (1). Such decline includes carnivores (2) and the charismatic tiger (*Panthera tigris*) in which four subspecies have become extinct in the last century (3). India is home to two-thirds of the world’s tigers, and protection, conservation, and monitoring suggest conservation gains (4). While tigers may have recovered in India overall, some populations remain small and isolated (5). Small and isolated populations have low genetic variation (6) and a high probability of fixation of deleterious alleles (5, 7, 8) because of inbreeding, demographic stochasticity, and random genetic drift, making them prone to extinction (9, 10).

Genetic drift can result in the fixation of a deleterious genetic variant over another neutral or even beneficial allele (11). The evidence for drift comes from differences in allele frequencies between replicate populations (12) or changes in allele frequency over time (13) in small populations. Few genetic studies of small, isolated, and endangered populations characterize differences in frequencies of particular variants, especially those associated with visible phenotypes (12–15). In this paper, we identified a genetic variant that causes a phenotypic change in tigers and quantified its frequency in several wild tiger populations, including one that is potentially small and isolated. We further investigated the role of drift in the observed frequency distribution across the tiger range.

Diverse pigmentation phenotypes that vary geographically have been observed in many species, including birds, butterflies, mice, cats, horses, and humans (16–25). Unique pigmentation patterns have also been observed [e.g., erythristic leopards (26), the albino fishing cat (27), the white-phased spirit black bear (28), the leucistic Antarctic fur seal (29), and leucistic dolphins (30)]. Together, these observations suggest that alleles responsible for pigmentation phenotypes should vary geographically and be impacted by gene flow and drift. While some studies have attempted to quantify drift using changes in phenotypic frequencies over time (12, 13), we should ideally investigate geographic variation in the frequency of the underlying genetic variant (18). This is often challenging in natural populations because our ability to link genotype to phenotype in non-model systems remains poor (31). Such studies are also plagued with issues of small sample size (29) and poor accessibility to biological material (29) in endangered species.

Tigers have a distinctive dark stripe pattern on a light background, which can appear in several color shades—white, golden, and snow white. Segregation of these color variants in captive tiger populations has permitted their genetic and molecular characterization (32, 33). A rare pattern variant, distinguished by pattern elements that are broadened and fused together, has also been observed in natural and captive tiger populations. Such tigers are sometimes called black tigers (34) (Fig. 1A and *SI Appendix*, Fig. S1), but the melanistic appearance is a consequence of expanded pattern elements rather than a uniformly darkened color, also referred to as pseudomelanism (35) and a term we use to describe the pattern morph henceforth. In the past, pseudomelanistic tigers have been reported from various places (*SI Appendix*, Table S1, reviewed in ref. 34). More recently, camera trap images from across global range have identified pseudomelanistic tigers from only one population (36), Similipal Tiger Reserve, Odisha (*SI Appendix*, Fig. S3), a 2,750-km^2^ protected area in eastern India. In addition to this wild population, pseudomelanistic tigers are present in three captive populations in India: Nandankanan Biological Park, Bhubaneswar (NKB), Arignar Anna Zoological Park, Chennai (AAC), and Bhagwan Birsa Biological Park, Ranchi, where they were born in captivity.

Distinct processes are involved in establishing and implementing mammalian color patterns (37). The implementation process occurs during recurring hair cycles and involves direct engagement with pigment cells to regulate light or dark pigment production, whereas the establishment process coordinates pattern formation during embryogenesis. *Taqpep* mutations in the domestic cat (*Felis catus*) and the cheetah (*Acinonyx jubatus*) (38) alter pattern formation in a manner that is strikingly similar to pseudomelanistic tigers, implicating *Taqpep* as a strong candidate for pseudomelanism in tigers (39).

In this paper, we confirmed a *Taqpep* missense mutational variant as the genetic basis for a rare pseudomelanistic phenotype in tigers using whole-genome sequence data and known pedigrees of captive tigers that included pseudomelanistic individuals. We confirm the presence of this mutation only in Simlipal, where it occurred at a high frequency in a sample of wild tigers from across their global range. Finally, we used population genetics analyses to investigate whether genetic drift may be responsible for the observed discordant frequency within and outside Similipal by 1) investigating whether Similipal is a small and isolated population and 2) conducting population genetic simulations to explore how founding bottlenecks and genetic drift may change allele frequencies.

## Results

### What Causes Pseudomelanism in Tigers?

**Fig. 1:**
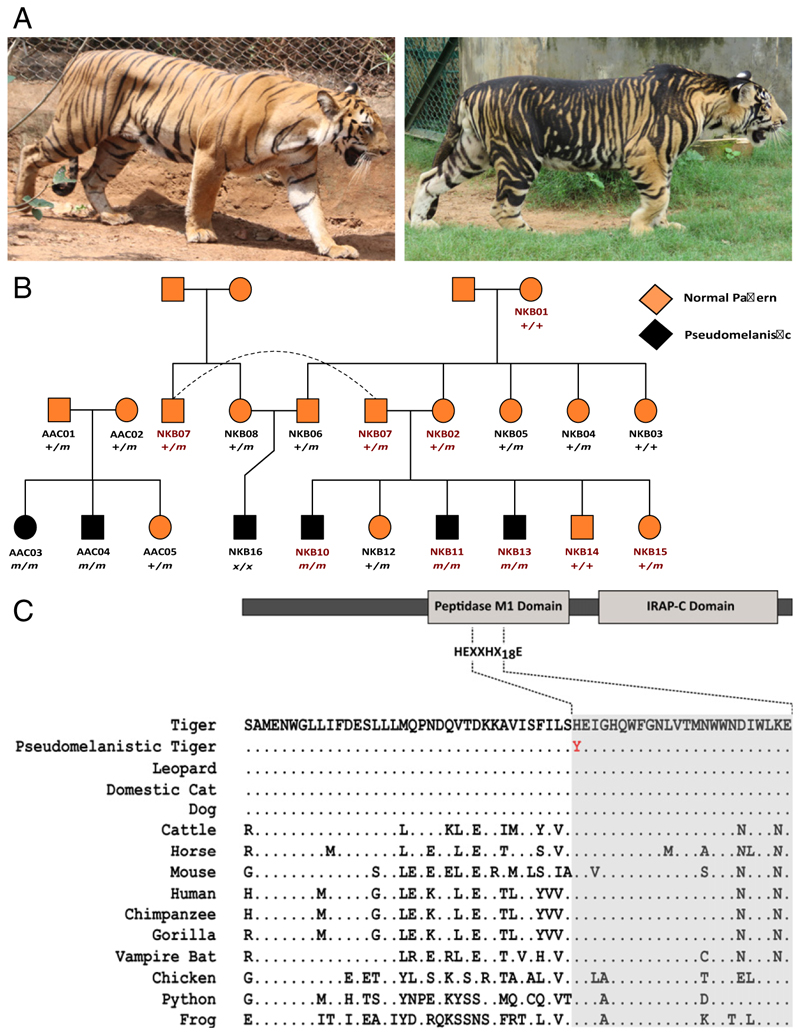
Identifying the genetic basis of pseudomelanism in captive tigers. (*A*) Normal tiger (*Left*) versus pseudomelanistic tiger (*Right*). An increase in the surface area of the coat covered by darker stripes gives the pseudomelanistic tiger a darker, blotchy appearance. (*B*) The pedigree of the captive tigers sampled for this study. The individual labels shown in red are for the tigers whose genome was sequenced for this study (NKB17 is not shown in the pedigree). The genotype values are indicated for the individuals sampled and successfully genotyped at the mutation site (+/+ for wild-type homozygote, +/*m* for heterozygote, *m/m* for mutant homozygote, and *x/x* for missing genotype). Squares represent males, and circles represent females. Pseudomelanistic phenotype is represented in solid black shapes. The dashed line shows the presence of the same individual at two spots in the pedigree. (*C*) Schematic diagram and partial alignment of Taqpep protein showing the HEXXHX_18_E motif (shaded) evolutionarily conserved among vertebrates. The histidine residue at position 454 is substituted by a tyrosine residue in the pseudomelanistic tigers (shown in red). This *Taqpep* mutational variant is distinct from the *Taqpep* causal variants implicated for domestic cat Tabby and King cheetah phenotype reported by Kaelin et al. (38)s

Pseudomelanism in tigers is inherited as an autosomal recessive trait as predicted from the captive pseudomelanistic tigers’ pedigree (Fig. 1B). Our analyses of the genomes of eight captive tigers (three pseudomelanistic) from NKB revealed that all the pseudomelanistic tigers were homozygous for a single nucleotide variant predicted to alter a conserved and functionally important residue in the *Taqpep* gene. The *Taqpep* gene in tigers has 25 exons, with the total coding sequence length being 3,093 base pairs [Assembly PanTig1.0(GCF_000464555.1)]. We observed a single base substitution in exon nine from C to T at position 1360 (*Taqpep* c.1360C > T), which translates into a missense mutation at position 454 (histidine to tyrosine) in the primary sequence (National Center for Biotechnology Information [NCBI] Reference Sequence: XP_007086933.1) of the protein (Taqpep p.H454Y) (Fig. 1C). His454 in tigers is the first histidine residue in the characteristic and highly conserved HEXXHX_18_E motif required for metal ion binding (Fig. 1C). We observed no other nonsynonymous substitutions in *Taqpep* that segregate with pseudomelanism. We genotyped noninvasive samples of eight additional individuals related to the pseudomelanistic tigers from NKB and five individuals (two pseudomelanistic) from AAC (same pedigree, *SI Appendix*, Table S3 and Fig. S2A) at the *Taqpep* c.1360C > T variant site and observed complete concordance of Taqpep p.H454Y with pseudomelanism [log likelihood test (LOD) = 2.5]. Loss-of-function mutations in *Taqpep* in other felid species (different positions) result in similar pattern variants (38), providing strong evidence for causality.

### What Is the Geographical Distribution of Taqpep p.H454Y Variant in Natural Populations?

#### Taqpep p.H454Y is present in tigers in Similipal Tiger Reserve

We observed the presence of the Taqpep p.H454Y allele in Similipal (Table 1). Among the 12 unique individuals that we identified in Similipal (from noninvasive samples—Methods and Results), we genotyped two wild-type homozygotes, four mutant homozygotes, and six heterozygotes (2+/+, 6+/*m*, and 4*m/m*) (Table 1 and *SI Appendix*, Fig. S3*B*), resulting in a Taqpep p.H454Y allele frequency of 0.58 in Similipal.

#### Taqpep p.H454Y allele frequency across tiger range

**Fig. 2:**
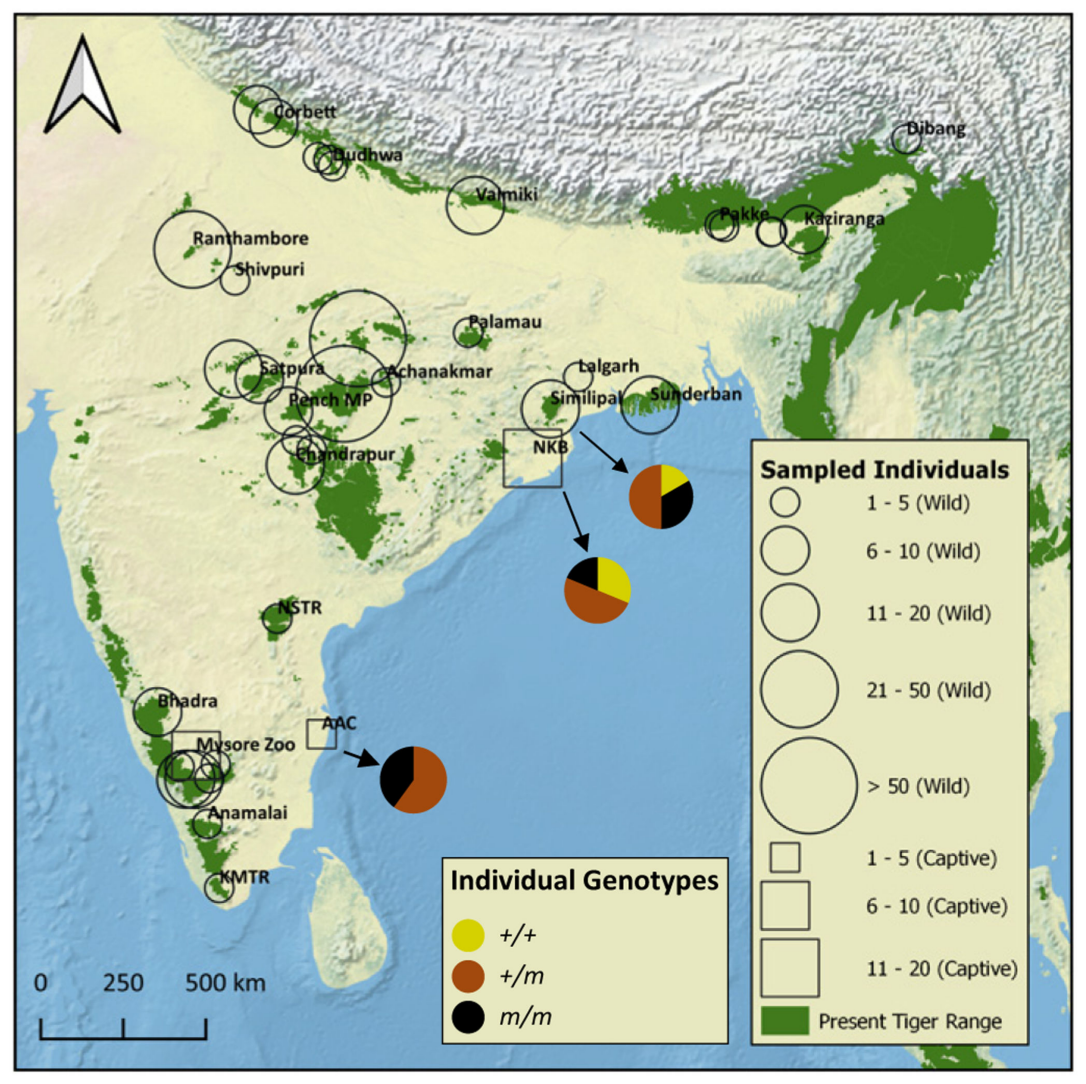
Distribution of the genotyped individuals. A total of 428 individuals were genotyped at the *Taqpep* c.1360C > T mutation site. Wild tigers are shown with a circular marker, and captive tigers (NKB, AAC, and Mysore Zoo) are shown with a square marker. The size of the square/circle indicates the number of individuals genotyped from a given area. In addition to the 399 Bengal tigers shown on the map, we genotyped 12 Amur, 12 Malayan, and five Sumatran tigers from Armstrong et al. (40) These are not shown on the map to allow the figure to focus on sampling within India. The fraction of the three genotypes in samples from the three populations in which pseudomelanistic tigers are present is shown with the pie chart. Similipal is the only population of wild tigers to have pseudomelanistic tigers, and the other two populations are of captive tigers. All wild tigers were homozygous for the wild-type allele at *Taqpep* c.1360C > T site except for Similipal individuals.

We studied 599 tigers across the tiger range at the *Taqpep* c.1360C > T variant site. We genotyped 85 tigers using whole-genome data for four subspecies [Amur, Malayan, and Sumatran: 29, Armstrong et al. (40), and Bengal tigers: 56, Khan et al. (5), Khan et al. (41), Armstrong et al. (40), and this study] and recorded all of them as homozygous for the wild-type allele. We also attempted to PCR amplify the *Taqpep* c.1360C > T mutation locus (Methods) from 528 genetically identified individuals from primarily noninvasive sources [samples from Natesh (42), Reddy et al. (43), Reddy et al. (44), and ongoing studies in Central India, North, and northeast India]. Of these, 309 individuals (58.5%) were successfully genotyped and were homozygous for the wild-type allele. In total, 395 tigers outside of Similipal, NKB, and AAC were homozygous for the wild-type allele at *Taqpep* c.1360C > T variant site (Fig. 2). Beyond these samples, we studied 330 noninvasive samples that could not be assigned to individuals because they did not produce enough single nucleotide polymorphism (SNP) data for individual identification. All 52 samples that we successfully genotyped were homozygous for the wild-type allele. Our results indicate that Taqpep p.H454Y is likely absent or extremely rare outside of Similipal.

### Population Genetics of Similipal Tigers—Small Population Size and Reduced Connectivity

#### Genetically identifying individuals from fecal samples

We collected a total of 137 noninvasive samples from Similipal in two sampling sessions spaced 1 y apart. Of these, we detected tiger-specific DNA in 62 samples (details of other samples in *SI Appendix*, Table S2). Nine samples failed to give any result for species identification.

We identified unique individuals by eliminating recaptures of the same individual after genotyping them at 126 SNP loci as described in Natesh et al. (45) (Methods). More than half (82) of the loci were removed during the filtering process (genotype quality [GQ] < 10, depth [DP] < 10, and minor allele count [MAC] = 1; Methods). We identified 12 unique individuals in Similipal (*SI Appendix*, Fig. S3*B*) based on the pairwise relatedness (PI-HAT) (46) values for all sample pairs based on 44 polymorphic neutral nuclear loci [probability of identity for two randomly chosen individuals, PID = 4.9E-16; probability of identity for two randomly chosen siblings, PID-sibs = 1.2E-8, allele frequencies based on genomes of 40 wild Indian tigers (5, 40, 41)].

#### Population structure and landscape connectivity

**Fig. 3:**
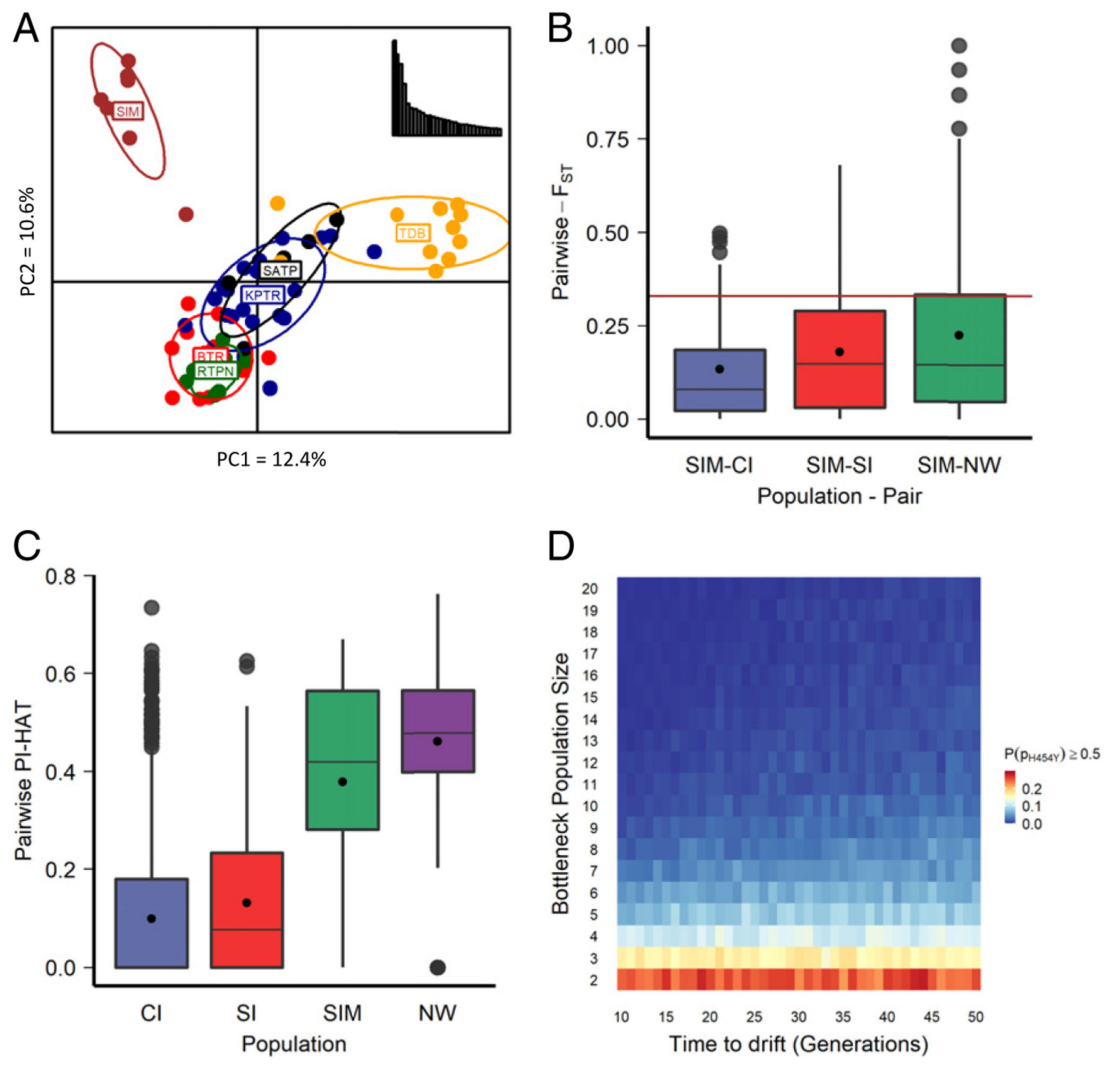
Population genetic analyses of Similipal tigers suggest genetic differentiation of Similipal from other tiger populations, indicating ongoing drift, and simulations suggest past bottlenecks might be responsible for a high frequency of Taqpep p.H454Y in Similipal. (*A*) Principal component analysis of Central Indian tiger populations [Kanha (KPTR; *n* = 22); Bandhavgarh (BTR; *n* = 13); Tadoba (TDB; *n* = 11); Satpura (SATP; *n* = 5); Ratapani (RTPN; *n* = 8)] including Similipal (SIM; *n* = 8) using data on 81 SNPs. Similipal separates out from other tiger populations on the first principal component (12.4%) and the second principal component (10.6%). (*B*) Box plot showing the distribution of pairwise Wright’s F_ST_ between Similipal (SIM) and three genetic clusters, namely, CI, SI, and NW, for 81 neutral loci including the *Taqpep* c.1360C > T mutation site. The central line of the box is the median, and the black dot is the mean value. The brown line indicates the value of F_ST_ for the *Taqpep* c.1360C > T site. (*C*) Distribution of relatedness (PI-HAT) between a pair of individuals in Similipal compared with the three genetic clusters. Similipal individuals show high relatedness and appear inbred like the NW population. (*D*) Heat map showing the probability of a mutant allele reaching a frequency ≥0.5 in an isolated population recovering after bottleneck under the effect of drift assuming one heterozygote in the founding population. The *x*-axis shows the time allowed to drift, and the *y*-axis shows the bottleneck population size.

Eight out of 12 tigers from Similipal were genotyped on 85 loci (out of 126; Methods), 81 of which were in Hardy−Weinberg equilibrium (HWE) and were retained (Methods) for further analyses. Previous studies have detected three major genetic clusters within Indian tigers—Central India, South India, and Northwest India (47). We observed that Similipal is genetically distinct from other Central Indian (*n* = 5 to 22) populations in a principal component (Fig. 3A) and population structure (48) analysis (*SI Appendix*, Fig. S4). Simlipal tigers form a separate genetic cluster at K = 3 (best K = 5). On average, Northwest India showed the highest differentiation from Similipal, followed by South India and then populations in Central India (Fig. 3A and Table 2 and *SI Appendix*, Fig. S4).

There are five small (200 to 800 km^2^) protected areas close to Similipal (100 to 700 km) where tiger presence was detected recently (as per forest department records). We conducted ground surveys in all five of these protected areas to collect noninvasive samples of tigers (Methods and *SI Appendix*, Fig. S3*A*). Only two fecal samples collected from these regions had tiger DNA (350 and 600 km away from Similipal), but neither yielded SNP genotype data.

We found significant support for isolation by distance (*P* value = 0.001; *SI Appendix*, Fig. S5*A*) but only at shorter geographic distances. A Mantel correlogram (*SI Appendix*, Fig. S5*B*) of genetic distance with geographic distance shows that isolation by distance breaks somewhere between 100 and 350 km.

Our least-cost resistance kernel analysis with dispersal thresholds of 200, 500, and 1,000 km suggests that the strength of the connectivity between Simlipal and the closest source populations was strong only at 1,000 km, weak at 500 km, and zero at 200 km dispersal threshold (*SI Appendix*, Fig. S5 *C−F*). In other words, if tigers can disperse 1,000 km, then Similipal will be connected to multiple nodes; if they can disperse 500 km, then one node. Similipal will be disconnected at a dispersal threshold of 200 km.

### Selection and inbreeding

The pairwise Wright’s F_ST_ at the *Taqpep* c.1360C > T variant site between Similipal and three previously identified genetic landscapes (Northwest India, South India, and Central India) was 0.33 (constant value because the wild-type allele is fixed in all three genetic landscapes). Pairwise F_ST_ based on 81 loci (*Population Genetics Analyses*—*Wild Tigers*) revealed that several loci [eight in Similipal—Central India (but highest pairwise Weir and Cockerham’s F_ST_), 15 in Similipal—South India, and 23 in Similipal—Northwest India] had higher levels of genetic differentiation than the *Taqpep* c.1360C > T variant (Fig. 3B), suggesting that the observed genetic differentiation at this site, though high, is not necessarily an outlier. The global F_ST_ value for the *Taqpep* c.1360C > T variant was among the top four loci out of these 81 loci (*SI Appendix*, Fig. S6*A*).

We cannot infer the absence of selection and selective advantage of the mutant allele because of the small number of loci in our data. However, there is no indication of deviation from HWE at this site in Simlipal based on Fisher’s exact test (*P* value = 1, *SI Appendix*, Table S6).

The mean observed heterozygosity in Similipal (mean H_o_ = 0.28) is reduced to similar levels as Northwest India (mean H_o_ = 0.22) as opposed to Central India (mean H_o_ = 0.36) and South India (mean H_o_ = 0.32) (*SI Appendix*, Fig. S6*B*), suggesting also that Similipal tigers are inbred. Mean relatedness between Similipal individuals was 0.38, close to the mean relatedness in another isolated and inbred population, (5) Northwest India (0.46), in contrast, to mean relatedness within Central India (0.09) and South India (0.13) (Fig. 3C).

### Simulations to Model Population History and Future Trajectory

Our noninvasive samples and the resulting low number of markers do not allow us to estimate the demographic history of Similipal tigers. Instead, we used simulations to investigate possible past trajectories and future evolutionary outcomes of *Taqpep* c.1360C > T in Similipal tigers. Our analyses thus far do not implicate selection at the *Taqpep* c.1360C > T variant site, so simulations only investigated possible scenarios under drift. Simulations of historical bottlenecks [assumed to be around the times of bounty hunting (49) and the beginning of habitat fragmentation (50)] and associated genetic drift (non-overlapping, discrete generations) suggest that extreme bottleneck scenarios may result in a substantial probability of the mutant allele frequency reaching observed levels [P(*p*_H454Y_ ≥ 0.5)]. For example, an isolated population recovering over 44 generations from a severe bottleneck (*n* = 2) results in the value of P(*p*_H454Y_ ≥ 0.5) = 0.28, assuming one heterozygote exists in the founding bottlenecked population (Fig. 3D). This was the highest value of P(*p*_H454Y_ ≥ 0.5) among several cases [P(*p*_H454Y_ ≥ 0.5) = 0 to 0.28] of bottleneck size (2 to 20 individuals) and time allowed for drift (10 to 50 generations). In other words, the timing of the bottleneck did not sub-stantially affect the probability of achieving a high frequency of Taqpep p.H454Y (see a low range of SDs in *SI Appendix*, Fig. S7*A*). Intense bottlenecks were required for any appreciable value of P(*p*_H454Y_ ≥ 0.5) (*SI Appendix*, Fig. S7*A*)). Corresponding simulations (same bottleneck size and time) had a lower value of P(*p*_H454Y_ ≥ 0.5) when we did not force the founders to include at least one Taqpep p.H454Y allele (*SI Appendix*, Fig. S5*B*), implicating founding events.

We investigated future evolutionary trajectories for this mutation with and without genetic rescue. Simulations were used to investigate the time required to fix either the mutant or the wild-type allele under different population growth and demographic scenarios. We observed that with complete isolation, the mean time required to fix the mutant allele is only 10.6 generations (2,000 replicates) in a constant population size of 10 individuals (no intrinsic growth) and a mutant allele frequency of 0.5 (*SI Appendix*, Fig. S7*C*). However, with logistic growth of the population [*r* = 0.03 per year (51)], the time to fixation increases with carrying capacity (mean time to fixation = 43.8 generations for K = 35 versus mean time to fixation = 102.3 generations for K = 104; 2,000 replicates; Methods) (52). The mutant allele gets fixed only 50% of the time as expected by theory (11). However, when one wild-type homozygous individual is introduced into the population every generation (~5 y), the wild-type allele gets fixed 100% of the time with a loss of the mutant allele. The time to fixation is also reduced (mean time to fixation = 9.1, 33.7, and 76.9 generations for the case of no intrinsic growth, logistic growth with K = 35, and logistic growth with K = 104, respectively) in the case of such assisted migration.

## Discussion

### A Novel Mutation for Pseudomelanism in Tigers

India’s pseudomelanistic tigers represent a unique opportunity to understand the genetic basis of morphologic variation in a rare and elusive endangered species. They are found in one wild population and three captive populations (in all captive populations, they were born in captivity, *SI Appendix*, Table S1). All captive pseudomelanistic tigers have ancestral links to one individual from Similipal that may have introduced Taqpep p.H454Y into the zoo populations (*SI Appendix*, Fig. S2*A*). Most of these captive tigers have mixed ancestry, but for the ones in Nandankanan zoo (closest to Similipal), a large part is from Central Indian tigers (*SI Appendix*, Fig. S2 *B* and *C*). Therefore, partial evidence points to Similipal being the source population of the Taqpep p.H454Y allele in the captive tiger populations of India.

Using whole-genome data of captive pseudomelanistic tigers, we identified a missense mutation in the *Taqpep* gene that is present in pseudomelanistic tigers in a recessive state. Taqpep is a member of the M1 family of metalloproteases which bind to a single Zn^2+^ ion for the Zn^2+^/water hydrolysis of the substrate (53). These proteins have a signature HEXXHX_18_E motif in which the two histidine residues and the distal glutamate residue are involved in Zn^2+^ ion binding (53–55) (reviewed in ref. 56). The proximal glutamate is required for water hydrolysis of peptide bonds and the subsequent release of the substrate (54, 57). His454 is the first histidine of the essential HEXXHX_18_E motif, and this residue is conserved among vertebrates (Fig. 1C). As the residue is essential for the catalytic activity of the protein (55), H454Y may result in reduced or no activity of the protein. Histidine to tyrosine sub-stitutions at homologous positions in other M1 family aminopeptidases have been reported to cause complete loss of catalytic activity (53, 58). Further studies involving functional and biochemical assays may shed more light on the impact of H454Y on the functionality of the protein.

Several recessively inherited *Taqpep* variants alter tabby markings in domestic cats and king cheetah (38). Our study highlights that *Taqpep* is a target for recurrent mutations in felids, likely due to the important function of other patterning components. Although we did not perform whole-genome association studies [these may even be impossible in tigers given recommended sample sizes (59)], in light of the evolutionary significance of H454 residue [genomic evolutionary rate profiling (GERP) (60) = 5.81, combined annotation-dependent depletion (61) = 29.2] and similar effects of mutations in the *Taqpep* gene in other felids, we have a strong case for *Taqpep* c.1360C > T being the causal variant for pseudomelanism in tigers. Moreover, the loss of catalytic activity in other proteins of the M1 family with homologous mutations and a high GERP score [although the relationship between GERP scores and fitness cost should be interpreted with caution (62)] also suggest a potential fitness cost of the *Taqpep* c.1360C > T mutation.

### High Frequency of the Mutant Allele in Similipal Tiger Reserve

We estimated the *Taqpep* variant frequency based on careful genetic discrimination of unique individuals in the Similipal Tiger Reserve. Estimates of allele frequency tend to be biased when population sizes are small (63). The India-wide tiger census conducted in 2018 photo captured eight unique individuals, three of which were pseudomelanistic tigers (64). Given recessive transmission and assuming HWE, the census data predicts an allele frequency of ~0.6, similar to the allele frequency value (0.58) estimated from genetic data in our study (*n* = 12). Mark-recapture models based on camera trap data have been used to estimate frequencies of melanistic leopards [e.g., see Harihar et al. (65)]. To the best of our knowledge, the seamless integration of genetic data in demographic analyses does not exist so far. Future research based on statistically robust capture–recapture models, supplemented with genetic information, will provide more precise longitudinal allele frequency data.

### Is the Pseudomelanistic Mutation Private to Similipal Tiger Reserve?

The pseudomelanistic variant is not detected in tigers sampled from across their geographic distribution (*n* = 395, Indian = 366). While we could sample all the remaining landscapes of tigers across India and most of their range (except Indochina), the approximate proportion of the population sample varied [Northwest India—49%, Central India—19%, and South India—6%, based on the most recent population estimates from All India Tiger Census 2018 (64)]. Taqpep p.H454Y, if present in any population, is likely to be a rare allele, and intensive sampling would be required to rule out its presence. Our sampling in certain populations was intensive (e.g., Kanha Tiger Reserve [TR] ~73%, Ranthambore TR ~49%, and Bandhavgarh TR ~46%), and yet we did not detect Taqpep p.H454Y in any of these. Overall, we genotyped a significant fraction (~13%) of all wild Indian tigers (2,967) and only found the Taqpep p.H454Y mutant in Similipal. Further, pseudomelanistic tigers have not been sighted or camera trapped anywhere except Similipal in the past 30 y (34, 36). However, there are a few historic anecdotal records of pseudomelanistic tigers in India’s different parts (*SI Appendix*, Table S1), including Central Indian forests (34). Future sampling could focus on these areas and on landscapes where our current sampling has been poor, such as northeast India.

### Drift and Isolation in Similipal Tiger Reserve

The stark difference in the frequency of Taqpep p.H454Y between Similipal and all the other populations hints toward the absence of/reduced gene flow, which is confirmed by analysis of genetic differentiation, suggesting potential isolation of the Similipal tiger population. Tiger occupancy maps from the India-wide census conducted by the National Tiger Conservation Authority (Government of India) every 4 y since 2006 also show no source population of tigers close to Similipal (64, 66–68). Geographically, the closest source population to Similipal is ~800 km away (36), a distance much larger than the average home range of Bengal tigers (20 to 110 km^2^) (69, 70), the average dispersal distance (78 to 124 km) (71), and the maximal dispersal distance based on allometric scaling equations (500 km) (72). While these are just theoretical expectations, and an individual dispersal longer than 500 km is possible and has indeed been documented for tigers (73), it is very rare. So, the probability of dispersers from the closest source populations reaching Simlipal is very low as suggested by our least-cost resistance kernel analysis (*SI Appendix*, Fig. S5 *C−F*). Least-cost methods determine the lowest cumulative resistance to travel between source and destination, assuming that an animal has complete knowledge of the landscape and is likely to follow the shortest path based on the resistance through the landscape. However, animals would seldom follow these exact trajectories, making the probability of tigers from other source populations reaching Simlipal even lower.

Taken together, our various population genetic and connectivity analyses suggest the following: 1) Similipal is most closely related to, yet distinct from, Central Indian populations; 2) the minimum number of tigers (identified by genetic data, Table 1) in Similipal was low, with no source tiger populations close enough; and 3) landscape analyses identified a very low likelihood of geneflow at theoretical and average dispersal thresholds. In summary, we inferred that Similipal is a small and isolated population. Population genetic theory suggests strong effects of genetic drift in such small and isolated populations.

Rare sightings of pseudomelanistic (homozygous mutant) tigers across the country in the past corroborate that the Taqpep p.H454Y allele, although rare, did not originate in Similipal and was likely to be present in heterozygotes. Assuming historical time scales of about 200 y for the isolation of Similipal, the increase in the Taqpep p.H454Y allele frequency must have occurred in 50 or fewer generations. Our simulations also suggest that even if the Taqpep p.H454Y allele frequency was much lower before the isolation of Similipal, intense bottlenecks with just one hetero-zygote in the founding population could increase the allele frequency to observed levels with high probability under a simple drift model. Similipal has potentially been through recent bottlenecks due to the mass hunting and poaching of prey animals and regular forest fires (74). A few studies have invoked genetic drift as the driver of color polymorphism in natural populations, for example, in the northern leopard frog (14), manta ray (75), and candy-striped spider (12). Like these, our results suggest genetic drift is the major evolutionary force driving the frequency of pseudomelanism in Similipal.

### Inbreeding

The occurrence of anomalous phenotypes in natural populations may be associated with a loss of genetic diversity in bottlenecked or inbred populations (76, 77). For example, the anomalous fur phenotype and unusual “rope” tail were reported in the extensively inbred wolves of Isle Royale, MI, along with several other abnormalities such as cataract and syndactyly (78). A high frequency of recessive traits in small isolated populations raises the possibility of inbreeding (79). A high relatedness between individuals in Similipal (mean relatedness = 0.38, Fig. 3C), low average heterozygosity (mean H_O_ = 0.28, *SI Appendix*, Fig. S6*B*), and low individual diversity (average *F* = 0.33, *SI Appendix*, Fig. S6*C*) also imply inbreeding. Other small and isolated tiger populations show strong genomic signatures of inbreeding and high mutation load (5). Inbreeding has also been documented in other endangered species, along with high frequencies of deleterious traits. Such inbreeding could have consequences in the future as observed in several carnivore species [for example, gray wolves (78), Florida panthers (80), meerkat (81), and the Arctic fox (82), reviewed in Hasselgren and Noren (83)] with decreased individual fitness, resulting in a higher probability of extinction. An analysis of runs of homozygosity with whole-genome data could be used to establish the actual levels of inbreeding and mutational load in the population [for example, Khan et al. (5)]. Alternatively, a long-term study of pedigrees using intensive field sampling could provide insights.

### Other Possible Causes for High Mutation Frequency in Similipal

We infer the effect of genetic drift at the *Taqpep* c.1360C > T allele based on the observation of high genetic differentiation across loci, including *Taqpep* c.1360C > T, small population size, and potential isolation. However, the selection favoring pseudomelanistic individuals could also result in the differential frequency distribution of this mutant allele. Niche modeling suggests that the frequency of melanistic leopards is higher in darker tropical and subtropical forests than in drier open habitats (84). Pseudomelanistic phenotypes could be locally adaptive in Similipal (34), which is dominated by tropical moist deciduous and semi-evergreen closed-canopy forest (~93% forest cover within the core forest has density >40%) (85), with a relatively darker understory. In such habitats, darker coat color may confer a selective advantage for both hunting and avoiding hunting pressure (84, 86).

While an outlier test based on the 81 loci we genotyped fails to reject the null hypothesis (HWE) and does not support selection (*SI Appendix*, Table S5), this inference may be premature given our small SNP set. Outlier tests, meant to identify loci with statistically significant higher (or lower) differentiation, are typically the first step in identifying a candidate set of loci for signatures of selection. However, outlier tests can suffer from both Type I and Type II errors under a range of scenarios, including specification of population structure, isolation by distance patterns, the strength of selection, degree of population divergence, and low power associated with examining a small number of populations (87–90). Formal tests for selection depend on methods that usually rely on an analysis of long haplotype data (91, 92).

At present, our data [81 loci from very short read data <100 base pair (bp)] precludes us from making robust inferences from outlier tests or other tests of selection. However, future studies incorporating whole genomes or genome-wide data from Similipal should help confirm the role of genetic drift versus selection. Presently, genetic drift appears to be the most parsimonious explanation for the observed frequency of the variant in Similipal.

Contrastingly, the nature of the mutation (potential loss of function because of the loss of a critical conserved residue, GERP = 5.81) suggests that it could be deleterious. Moreover, while pseudomelanistic tigers occur at a high frequency in Similipal, they have disappeared from across India, where populations may be larger (and hence selection more effective). This lends support to the possible deleterious effects of Taqpep p.H454Y.

### Future Evolution and Conservation Implications for Tigers in Similipal

Conservation practice recommends the genetic rescue of populations such as Similipal that are small and isolated, with potentially related and inbred individuals (93). Our future simulations suggest that one migrant per generation would most likely result in the loss of the melanistic mutation from Similipal. On the other hand, no genetic rescue would cause fixation or loss of the mutant allele with a 50% probability in a relatively short period of time (*SI Appendix*, Fig. S5*C*). Regardless of how the frequency of this mutation changes in the future, genetic rescue should benefit the population by increasing heterozygosity and decreasing the probability of inbreeding depression (94). Careful consideration would be required when selecting the immigrant individuals. Ideally, such individuals could be from geographically proximate but high heterozygosity populations [see Khan et al. (5) for possible strategies]. Additionally, longer-term demographic and genetic studies within Similipal could help determine the fitness consequences of pseudomelanism to better understand whether changes in the frequency of this allele would impact population growth rates.

## Conclusions

Investigating the impacts of recent isolation and population size change on phenotypes remains difficult. This requires identifying the genetic basis for phenotypes, often difficult in non-model organisms, especially in endangered species in which mostly noninvasive samples may be available. Camera trap data reveal a high frequency of the pseudomelanistic phenotype in the Similipal Tiger Reserve, and our genetic data confirm that Similipal is small and isolated. We used whole-genome data from captive pseudomelanistic tigers to identify the genetic basis of this rare phenotype and characterized the frequency of this missense mutation within and outside Similipal. Our population genetic data and simulations suggest drift driven by recent bottlenecks and isolation is most likely responsible for the high local frequency of pseudomelanism in Similipal.

The pseudomelanistic tigers of Similipal present a rare case of rapid evolutionary change, with this allele possibly on its way to fixation. Managers are faced with a choice of fixation of the mutant allele and a need for genetic rescue strategies. Our study highlights the importance of inferring genetics of endangered species in the wild from a combined analysis of noninvasive samples from unknown wild individuals and blood/tissue samples from captive pedigrees. Finally, the high frequency of the pseudomelanistic tigers in Similipal and the apparent absence everywhere else suggests strong stochastic effects and inbreeding operating locally in this population.

## Methods

### Sample Collection

#### Captive tigers

We collected samples from two Indian zoological parks that house pseudomelanistic tigers, namely NKB (three pseudomelanistic tigers) and AAC (two pseudomelanistic tigers). We used whole-genome sequence data from captive pseudomelanistic tigers and their relatives of NKB (*n* = 9) to identify the causal genetic variant for the pseudomelanistic phenotype and targeted Sanger sequencing of individuals from NKB (*n* = 7) and AAC (*n* = 5) for genetic linkage analysis (*SI Appendix*, Table S2). We collected feces, shed hair, saliva, and blood samples from 22 captive tigers for this purpose. We collected the shed hair and fecal samples of the captive individuals from their cage before the scheduled early morning cleaning. To collect saliva samples from the captive tigers, we gave the isolated animal a clean PVC pipe to chew and then collected the salivary fluid from the pipe with a sterile swab (HiMedia). The blood samples of captive tigers used in this study were collected by the zoo hospital and Odisha University of Agricultural Technology for medical and research purposes. We stored the fecal and saliva samples in Longmire’s buffer (95) and the blood samples in ethylenediaminetetraacetic acid (EDTA) coated vials at −20 °C until DNA extraction from those samples. We acquired the information on the pedigree (Fig. 1B) of the captive tigers from the zoo studbook. All the samples were collected under the supervision of a zoo veterinarian with prior permission from the Central Zoo Authority (CZA), National Tiger Conservation Authority (NTCA), and Odisha State Forest Department (OSFD).

#### Wild tigers

To determine the frequency of the mutant allele in the wild, we collected noninvasive samples from six protected areas (PAs) in the state of Odisha, Similipal Tiger Reserve, Satkosia Tiger Reserve (SATK), Sunabeda Wildlife Sanctuary (SNBD), Debrigarh Wildlife Sanctuary (DEB), Hadgarh Wildlife Sanctuary (HADG), and Kuldiha Wildlife Sanctuary (KULD). We sampled Similipal over two seasons spaced 1 y apart (February to March 2018 and March 2019) and other PAs for one season (February to April 2019). Within each PA, we determined several 10- to 20-km sampling tracks based on the information provided by the forest staff. We walked along the tracks and collected fecal samples by swabbing over the surface with a sterile polyester swab (HiMedia) and storing the swab in Longmire’s buffer (95). We also collected some fecal samples, especially the dry and old ones, in ethanol as small (~200-gm) chunks. We collected shed-hair samples from scratch marks in the ground dry in zip lock bags. Finally, we collected saliva samples from the predation mark or lick mark on the prey body suspectedly killed by a tiger by swabbing over the surface and storing the swab in Longmire’s buffer. Within Similipal, the tracks were repeated after a minimum period of 6 d, depending on the informed tiger density/presence in the given area (*SI Appendix*, Fig. S3*B*). Since our objective was to maximize the sample size, we also collected some opportunistic samples (*n* = 27) based on the information from the forest guards on sighting a tiger’s feces. Within Similipal, we walked 346 km (21 tracks) twice and 124 km (14 tracks) once over 45 d in sampling season one and 20 d in sampling season two. We covered 164 km in SNBD (14 tracks), 261 km in DEB (17 tracks), 212 km in SATK (15 tracks), 41 km in HADG (4 tracks), and 56 km in KULD (4 tracks). We also collected two skin samples that were confiscated and stored at room temperature by OSFD. One skin sample was from a pseudomelanistic tiger that died in 1992 and the second from a normal tiger that died in 2015. The skin samples were collected dry and stored at room temperature on the field site. We stored all the samples at room temperature at the field site for 15 to 30 d until transferred to a −20 °C freezer in the laboratory. The sampling was conducted with prior permission from the NTCA and OSFD following forest department guidelines.

#### Samples and data from other sources

To survey different populations across the tiger range for the presence/absence of Taqpep p.H454Y and to increase our sample size for population genetics analyses, we used samples collected for other studies. The samples used in this study are from Reddy et al. (43), Reddy et al. (44), Natesh et al. (42), and ongoing studies in Central India, North, and northeast India. Additionally, we also used whole-genome sequence datasets from Armstrong et al. (40), Khan et al. (41), and Khan et al. (5) for our *Admixture* (96) analysis, to increase the sample size for our 81-SNP dataset (by subsetting data from whole-genome sequences) used for population genetics analysis, and to increase the sample size for *Taqpep* c.1360C > T genotyping.

#### DNA Extraction and Whole-Genome Sequencing

We extracted the DNA from the fecal swab, shed hair, saliva swabs, and blood samples using the Qiagen DNA Extraction Kit following the manufacturer’s instructions. We extracted the DNA from fecal chunks using the HiMedia Stool DNA Extraction Kit per the manufacturer’s protocol. We quantified the amount of DNA using Qubit (Invitrogen Qubit 3.0) and assessed its integrity based on the Bioanalyzer profile. The DNA extracts obtained from blood and saliva samples with high DNA integrity and concentration were selected for paired-end sequencing (20×; only NKB14 was sequenced at 5×) on the Illumina HiSeq 2500 platform. In total, we sequenced genomes from nine captive tigers: three pseudomelanistic individuals (siblings), their two parents, their grandmother, their two siblings, and one wild-caught, unrelated individual.

#### Identifying the Causal Mutation

We trimmed the whole-genome sequences using *TrimGalore* (97) for a quality threshold of 30 on the phred33 scale with a stringency value of 3. We aligned the resulting reads to the annotated domestic cat genome (felCat8.0 assembly; RefSeq accession: GCF_000181335.2) using *BWA-MEM* (98) with default settings and sorted the reads using *Sam-tools* (99). We marked the duplicate reads using *Picard Tools* (100). We utilized a candidate gene approach to find the genetic variants present in the *Taqpep* gene sequence of pseudomelanistic tigers. We subsampled our whole-genome alignment file for reads aligning to the *Taqpep* genomic DNA region (~96 kb) and called variants using *Freebayes* (101). The pedigree of the captive pseu-domelanistic tiger suggests that this phenotype is inherited in an autosomal recessive manner. We manually observed variants in the variant call format (VCF) file to identify one that matched the recessive inheritance pattern suggested by the pedigree to identify the potential causal mutation. We performed an LOD to assess the linkage of the identified mutation with the phenotype within the pedigree of captive tigers using *Merlin* (102). Because the combined pedigree of the pseudomelanistic tigers becomes too complicated and *Merlin* fails to detect the association (no genotype data for most individuals in the large pedigree given in *SI Appendix*, Fig. S2*A*), we assumed the pseudomelanistic tigers from NKB and AAC to be in two separate families.

#### Species Identification of the Noninvasive Samples and Individual Recaptures

We determined the species of the noninvasive samples collected from the wild using PCR amplification of a 202-bp region (primer sequence in *SI Appendix*, Table S4) of the 16S ribosomal RNA gene (denaturation at 95 °C, annealing at 56 °C, and elongation at 72 °C for 35 cycles) and sequencing the obtained products on a Sanger sequencing platform followed by an NCBI nucleotide BLAST of the sequences. To identify recaptures within the collected tiger samples from the wild, we performed SNP typing using multiplex PCR and MiSeq as described in Natesh et al. (45). Most noninvasive genetic sampling Downloaded from https://www.pnas.org by 49.206.5.172 on April 26, 2025 from IP address 49.206.5.172.

studies face the issue of poor DNA quality and low concentration, leading to erroneous genotypes (103). Allelic dropout at any polymorphic site, especially if two different alleles are read on the same heterozygous individual’s recaptures, can lead to entirely unrelated genotypes, eventually creating false individuals (103). We included technical replicates for our samples to avoid such errors when generating data on 123 polymorphic SNPs.

We trimmed the data obtained from the MiSeq run for adaptor sequences and low-quality reads using *TrimGalore* (97) for a quality value of 30 on the phred33 scale and a stringency value of 5. We aligned the retained reads to the reference Bengal Tiger Genome (BenTig1.0, NCBI accession: JAHFZI000000000) using *BWA-MEM* (98) with a mismatch penalty value of 3 and called variants using *bcftools* (99). We used *GATK* (104) to mark any genotype with a genotype quality value less than 10 (GQ < 10) and a depth less than 10 (DP < 10) as a missing genotype. We removed loci with missing data for more than 10% of samples and samples with genotype calls at less than 50 loci during the filtering using *VCFtools* (105). We also removed all the loci that were monomorphic within Similipal. Eventually, we used the genotypes at 44 loci for 53 samples. We calculated PI-HAT values among the samples using *Plink* (46). We observed that the same sample’s replicates had a minimum relatedness value of 0.78. Therefore, we marked any two samples with PI-HAT > 0.78 as recaptures of the same individual. Furthermore, we removed one sample of the pairs in which PI-HAT was 0.6 to 0.78 to ensure that a recapture is not identified as a separate individual. For sample pairs with PI-HAT < 0.6, we kept unique samples that were not already identified as recaptures as separate individuals. We calculated the PID (probability of any two individuals having identical genotypes) for the given loci using genotype data for 40 wild Indian tigers (40) using the *GenAlEx* (106, 107) plugin in Microsoft Excel. Individual identification for non-Similipal individuals from the noninvasive samples was carried out in similar ways as described for Similipal individuals with minor changes.

#### Genotyping at the Taqpep Mutation Site

Noninvasive samples often contain low-quality DNA, which can cause a large number of genotyping errors (103). To account for the errors and assess the collected noninvasive tiger samples’ genotype correctly, we used three different methods to genotype the samples. The first method included amplifying a short DNA fragment (161 bp) containing the mutation site using PCR (denaturation at 95 °C, annealing at 59 °C, and elongation at 72 °C; 40 cycles) followed by Sanger sequencing (success rate = 0.52), the second method involved adding mutation SNP-specific primers in the multiplex PCR primer panel described for individual identification and obtaining the genotype from next generation sequence (NGS) data (success rate = 0.6), and the third method involved amplifying specific alleles with allele-specific primers in a PCR (denaturation at 95 °C, annealing at 61.7 °C, and elongation at 72 °C; 35 cycles, for the wild-type−specific primers, and denaturation at 95 °C, annealing at 58 °C, and elongation at 72 °C; 35 cycles for the mutant-specific primers) and obtaining the genotype data from the gel image (success rate = 0.68). For allele-specific PCR (AS-PCR), we designed a common forward primer for both mutant and wild-type alleles and different reverse primers for each. The specificity was obtained by introducing a mismatch at the −2 position from the 3′ end (108). We confirmed most individual’s genotypes by at least two methods or from recaptures of the same individual. If any two methods assigned a different genotype to a sample, AS-PCR was repeated thrice for such samples [multiple tube approach (103)], and the final genotype was accepted only if the same genotype value was produced for all three replicates.

### Population Genetics Analyses—Wild Tigers

#### Data filtering

We identified 85 loci with ≤10% missing data among eight Similipal individuals (identified based on the genotypes at 44 SNPs). We did an exact test for the HWE at these 85 loci by subsetting the data into individual populations (according to protected area boundaries) in *Adegenet* (109) (version 2.1.3) and *Pegas* (110) (version 1.0−1) packages in R. Four loci from 85 were out of HWE at a significance level of 0.05 in two or more populations (out of eight). These loci were dropped from further analyses, thus leaving 81 loci for our population genetics analyses.

#### Genetic variation, differentiation, and isolation by distance

We estimated Wright’s pairwise F_ST_ between Similipal and three genetic clusters of tigers in India (47) at 81 loci for unique individuals, including the *Taqpep* variant site using *GenAlEx* (106, 107) (version 6.503). We extracted data on these 81 loci from a whole-genome dataset [generated for Armstrong et al. (40) and Khan et al. (5)] for the Bengal tigers to increase our sample size for Northwestern (NW) India, Central and North India (CI), and South India (SI) clusters. Overall (genomes and noninvasive samples combined), we used data for 15 tigers from NW India, 42 individuals from SI, 59 individuals from CI, and eight individuals from Similipal. We used this dataset to calculate the inbreeding coefficient for each individual (*F*) in *VCFtools* (it reflects the inbreeding level of an individual with respect to the total population) and plotted population-wise *F* by categorizing individuals into subpopulations. We estimated global and pairwise Weir and Cockerham’s F_ST_ using the *Pegas* (110) (version 1.0−1) package in R and observed and expected heterozygosity using *GenALEx* (106, 107) from this dataset. PI-HAT, a measure of relatedness between a pair of individuals, was estimated using *Plink* (*genome* function) (46) for this dataset after binning the data into four groups: Similipal, NW, SI, and CI.

We did a principal component analysis of this dataset using the *Adegenet* (109) (version 2.1.3) package in R. We did a population structure analysis of 59 Central Indian tigers and eight Similipal tigers by subsetting the same 81 loci dataset described in the earlier section using *Structure* (version 2.3.4) program (48). For *Structure*, we did two million Markov chain Monte Carlo repeats with a burn-in period of 50,000 for K = 2, 3, 4, 5, 6, 7, and 8, with 10 repeats for each value of K. The results of *Structure* were analyzed and plotted using the CLUMPAK (111) web-based tool.

We did isolation by distance analysis for 59 Central Indian tigers and eight Similipal tigers with genotype data on 81 loci using the *Adegenet* (109) (version 2.1.3) package in R. To obtain a Mantel correlogram, we used the *Vegan* (112) (version 2.5−7) package in R.

#### Ancestry of Captive Tigers

To understand the geographic origin of NKB tigers, we did an *Admixture* (version 1.3.0) (96) analysis of nine NKB tiger genomes with 50 wild Indian tiger genomes [wild tiger genomes from Armstrong et al. (40), Khan et al. (41), and Khan et al. (5)]. This was done for K = 2, 3, 4, 5, 6, 7, 8, 9, and 10 with 10 repeats of each K. The best K value was found using the −cv option while running the *Admixture* analysis as suggested in the program manual.

We also did a principal component analysis for captive tigers combined with wild tigers using the *Adegenet* (109) (version 2.1.3) package in R. For this, we merged genotype data at 81 SNPs from 124 wild Indian tigers (dataset described in Population Genetics Analyses—Wild Tigers) with 81 SNPs genotype dataset for the captive tigers. The data for AAC was obtained by mPCR (as described in *Species Identification of the Noninvasive Samples and Individual Recaptures*). The data for NKB individuals was subset from the whole-genome data described in *DNA Extraction and Whole-Genome Sequencing*.

#### Landscape Analyses

We used UNICOR (113) to produce factorial least-cost paths based on the resistance surface from Pariwakam et al. (114) with dispersal thresholds of 200, 500, and 1,000 km. The output depicts the location and strength of connections between nodes (PAs).

#### Simulations

To understand the role of drift in driving the allele frequency, we ran genetic drift simulations for an isolated population recovering after a bottleneck. We ran the simulations for a bottleneck population size of 2 to 20 and allowed time to drift 10 to 50 generations. This corresponds to 50 to 250 y before the present, assuming a generation time of 5 y (Anthrom data suggests most of the Central Indian tiger habitat declined within this period) (50). The simulated population was allowed to recover from the bottleneck under the logistic growth equation. The population growth rate was taken to be 0.03 annually (0.15 per generation) from Karanth et al. (51), and the carrying capacity was assumed to be 104 (52).

A founding population keeping only one heterozygote was assigned at the start of the simulation, and the allele frequency was calculated (*p_0_* = 1/2N_0_, where N_0_ is the starting population size). To build the next generation, a random number was generated between 0 and 1 using the *runif* function in R, and if the chosen random number was more than *p*_0,_ the wild-type allele was picked; otherwise, the mutant allele was picked. This was done N_0_ × 2 times to build the whole population. This process of random sampling was repeated for each generation. The last generation’s allele frequency was stored in a matrix, and the whole simulation was repeated 1,000 times for each value of bottleneck size and time to drift. We calculated the probability of Taqpep p.H454Y allele frequency reaching 0.5 and above (the observed frequency of pseudomelanistic allele in Similipal) by counting the number of times allele frequency crossed the 0.5 mark and dividing that by 1,000 and plotted this probability matrix as a heat map.

Future projection simulations to estimate time to fixation under two conditions—1) complete isolation of Similipal and 2) one wild-type homozygous individual is introduced into Similipal every generation (5 y)—were also done using a similar growth model (*r* = 0.15 per generation) (51) till one of the alleles gets fixed in the population. Two different carrying capacity values (K = 35 and K = 104) were used for logistic population growth model simulations as estimated from Upadhyay et al. (52) (multiplying the carrying capacity/100 km^2^ with Similipal total area 2,750 km^2^).

## Figures and Tables

**Table 1:** Genotypes of Similipal individuals at the variant site

S. No.	Individual Index	Recaptures		Sample Genotype			Individual Genotype
			Sanger	NGS	AS-PCR	Consensus	
1	INDV-1	STR18F-01		+/+	+/+	+/+	+/+
		STR18F-25	+/+		+/+	+/+	
		STR18F-75	+/+	+/+	+/+	+/+	
		SIM19F-14	+/+	+/+	+/+	+/+	
2	INDV-2	STR18-02	+/*m*		+/*m*	+/*m*	+/*m*
		STR18-27	+/*m*		+/*m*	+/*m*	
		STR18-49	+/*m*		+/*m*	+/*m*	
		SIM19-09		+/*m*	+/*m*	+/*m*	
		SIM19-29	+/*m*	+/*m*	+/*m*	+/*m*	
3	INDV-3	STRF18-04	+/*m*		+/*m*	+/*m*	+/*m*
		STRF18-19	+/*m*	+/*m*	+/*m*	+/*m*	
		STRF18-21	+/*m*		+/*m*	+/*m*	
		STRF18-43	+/*m*	+/*m*	+/*m*	+/*m*	
		STRF18-56	+/*m*		+/*m*	+/*m*	
		STRF18-57		+/*m*	+/*m*	+/*m*	
		STRF18-58			+/*m*	+/*m*	
		STRF18-81	+/+		+/*m*	+/*m*	
		STRF18-92	+/*m*	+/*m*	+/*m*	+/*m*	
4	INDV-4	STRF18-07	*m/m*	*m/m*	*m/m*	*m/m*	*m/m*
		STRF18-20	*m/m*	*m/m*	*m/m*	*m/m*	
		STRF18-26	*m/m*	*m/m*	*m/m*	*m/m*	
		STRF18-41	*m/m*	*m/m*	*m/m*	*m/m*	
		STRF18-46		*m/m*	*m/m*	*m/m*	
		STRF18-61	*m/m*	*m/m*	*m/m*	*m/m*	
		STRF18-84	*m/m*		*m/m*	*m/m*	
5	INDV-5	STRF18-09		**+/*m***	*m/m*	*m/m*	*m/m*
		SIMF19-01	*m/m*	*m/m*	*m/m*	*m/m*	
6	INDV-6	STRF18-10			***m/m***	***m/m***	+/*m*
		STRF18-11	+/*m*	+/*m*	+/*m*	+/*m*	
		STRF18-50	+/*m*	+/*m*	+/*m*	+/*m*	
		STRF18-94	+/+		+/*m*	+/*m*	
7	INDV-7	STRF18-08			*m/m*	*m/m*	*m/m*
8	INDV-8	STRF18-59	+/+		+/+	+/+	+/+
9	INDV-9	STRF18-62	m/m	*m/m*		*m/m*	*m/m*
10	INDV-10	STRF18-85			+/*m*	+/*m*	+/*m*
11	INDV-11	SIM18T-103	+/m			+/*m*	+/*m*
12	INDV-12	SIM19-08	+/m	+/*m*	+/*m*	+/*m*	+/*m*

The genotypes as obtained by all three genotyping methods are listed for each sample. Blank spaces indicate the instances in which a particular method failed to provide a genotype. Bold face letters indicate the wrong genotype calls as confirmed by recaptures. The consensus genotype was decided based on the frequency of the same genotype by different methods, and the final genotype for the individuals was decided based on the frequency of the same genotype in recaptures, if any. Abbreviations: NGS: next-generation sequencing, AS-PCR: allele-specific PCR.

**Table 2:** Pairwise F_ST_ values between populations using genotypes at 81 loci

CI	NW	SI	SIM	
0.000				CI
0.172	0.000			NW
0.075	0.220	0.000		SI
0.133	0.246	0.191	0.000	SIM

Abbreviations: C: Central India, NW: Northwest India, SI: South India, and SIM: Similipal.

## Data Availability

Raw sequence data have been deposited in NCBI (Bioproject accession no. PRJNA749163). Previously published data were used for this work (https://doi.org/10.1093/molbev/msab032, https://doi.org/10.1002/ece3.6157, and https://doi.org/10.1101/2021.05.18.444660). Scripts for variant calling and filtering, population genetics simulations, and datasheets are available from Github (https://doi.org/10.5281/zenodo.5244876).

## Significance

Small and isolated populations have low genetic variation due to founding bottlenecks and genetic drift. Few empirical studies demonstrate visible phenotypic change associated with drift using genetic data in endangered species. We used genomic analyses of a captive tiger pedigree to identify the genetic basis for a rare trait, pseudomelanism, in tigers. Genome sequencing and extensive genotyping of noninvasive samples across tiger range revealed unique spatial presence of this allele in the Similipal Tiger Reserve, India. Population genetic analyses confirmed that Similipal is a small and isolated population. Simulations suggest that intense founding bottlenecks could result in the observed patterns, implicating drift. Our study highlights ongoing phenotypic evolution, potentially from human-induced fragmentation, in endangered large carnivore populations.
