# Supplementary material for: High frequency of an otherwise rare phenotype in a small and isolated tiger population
Source: Proc Natl Acad Sci U S A. 2021 Sep 13;118(39):e2025273118. doi: 10.1073/pnas.2025273118 (PMC8488692; doi:10.1073/pnas.2025273118)
Supplement: Supplementary file 1 [file pnas.2025273118.sapp.pdf]

PNAS
www.pnas.org

Supplementary Information for

High frequency of an otherwise rare phenotype in a small and isolated tiger population

Vinay Sagar
Christopher B. Kaelin
Meghana Natesh
Anuradha Reddy
Rajesh K. Mohapatra
Himanshu Chhattani
Prachi Thatte
Srinivas Vaidyanathan
Suvankar Biswas
Supriya Bhatt
Shashi Paul
Yadavendradev V. Jhala
Mayank M. Verma
Bivash Pandav
Samrat Mondol
Gregory S. Barsh
Debabrata Swain
Uma Ramakrishnan

Uma Ramkrishnan, Vinay Sagar
Email: uramakri@ncbs.res.in, vinays@ncbs.res.in

This PDF file includes:

Figures S1 to S7
Tables S1 to S6
SI References

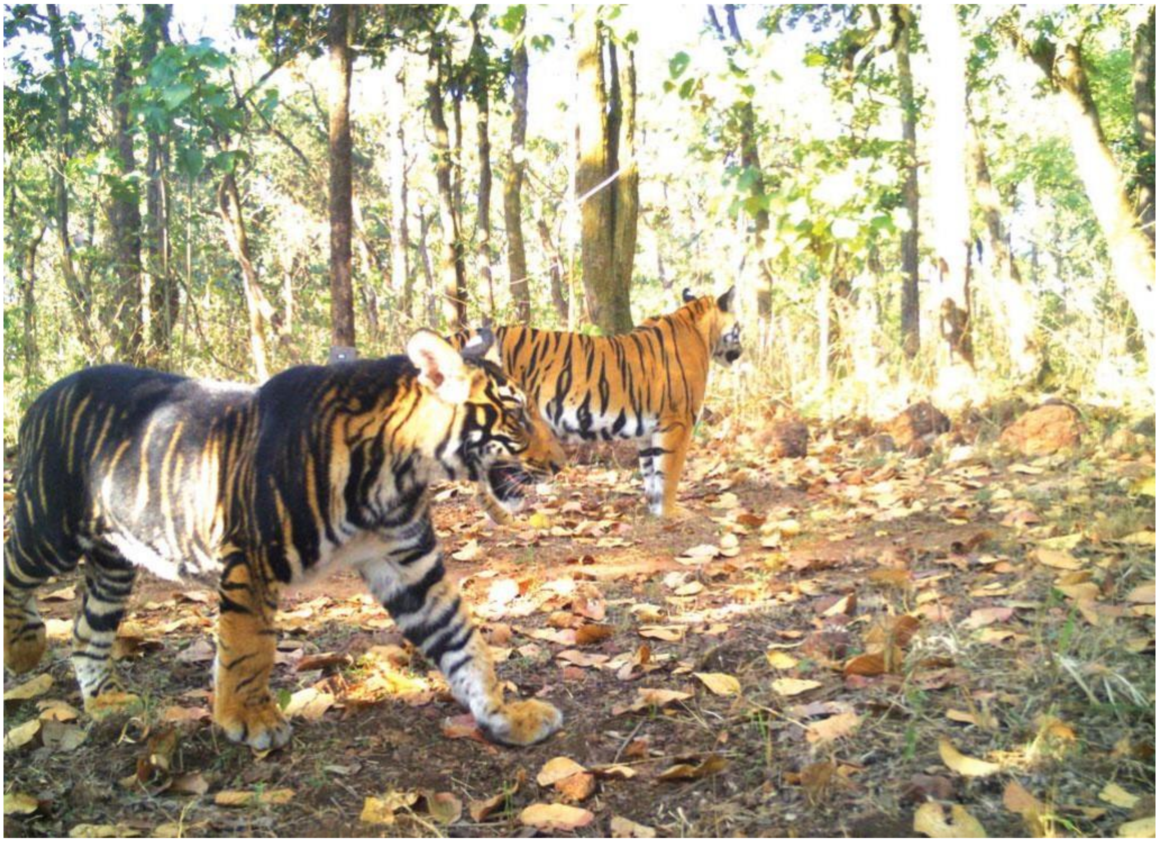

Fig. S1. Camera trap image of a pseudomelanistic and a normal tiger inside Similipal Tiger Reserve

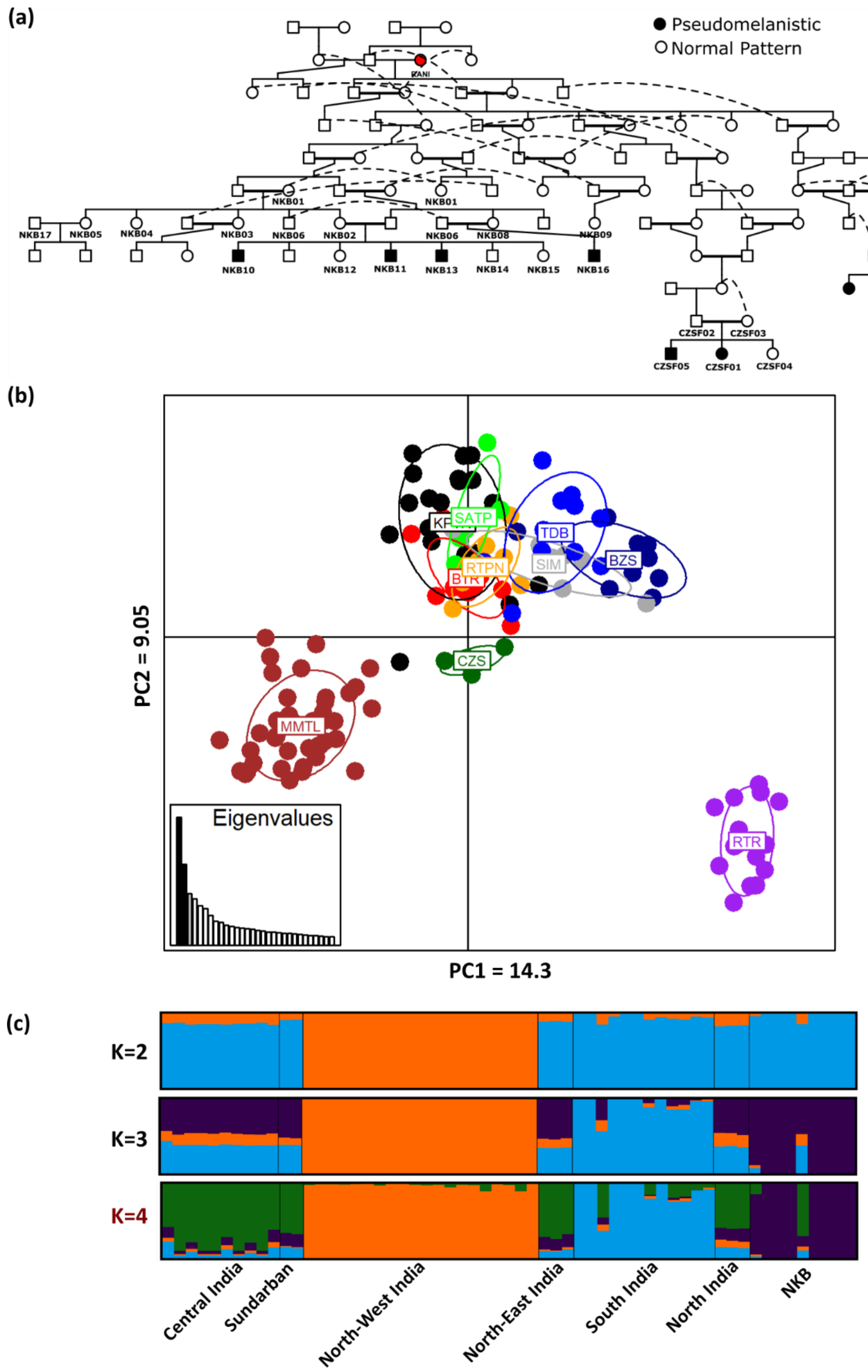
(a)
Pseudomelanistic
Normal Pattern
(b)
KP
SATP
TDB
BZS
SIM
RTPN
BTR
CZS
MMTL
RTR
Eigenvalues
PC2 = 9.05
PC1 = 14.3
(c)
K=2
K=3
K=4
Central India
Sundarban
North-West India
North-East India
South India
North India
NKB

Fig. S2 – Geographic origin of captive pseudomelanistic tigers – (a) A combined pedigree of pseudomelanistic tigers from all three Indian zoos. All captive pseudomelanistic individuals have possible ancestral links to a wild-caught individual from Similipal (shown in red here). The individuals sampled in this study are labelled with their sample IDs (b) A principal component analysis of 124 wild tigers and captive tigers from NKB and AAC with genotype data on 81 loci. NKB (marked as BZS here) tigers cluster close to Similipal and other central Indian tiger populations. AAC (marked as CZS here) cluster between central Indian and South Indian (marked as MMTL here) tigers. (Abbreviations used: BZS – NKB, CZS – AAC, MMTL – South India, RTR – North West, SIM – Similipal, TDB – Tadoba,, KPTR – Kanha Pench Tiger Reserve, BTR – Bandhavgarh, RTPN – Ratapani, SATP – Satpura and (c) Admixture analysis of captive tiger genomes of NKB along with individuals from several parts of India using genotype data on 2.9 million SNPs. NKB tigers form a separate cluster at K = 3 and derive their ancestry from central Indian tigers. At the best K value of 4 (shown in red), NKB tigers appear to be differentiated from central Indian tigers.

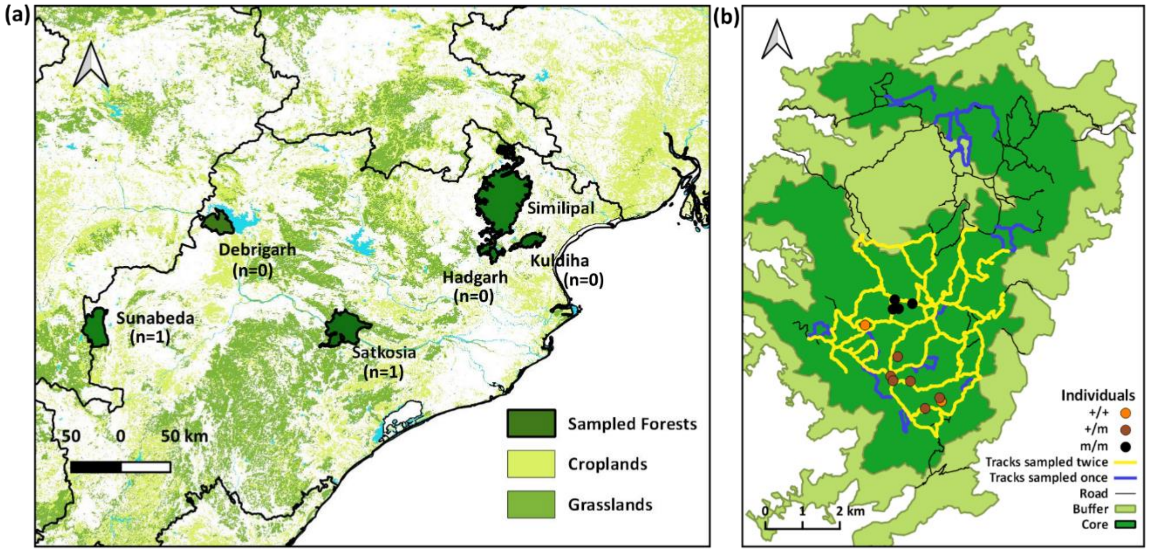

Fig. S3 – Non-invasive samples collected for this study (a) Five protected areas close to Similipal were sampled. The number of tiger samples collected from each protected area is shown in parenthesis (b) Geographical distribution of identified unique individuals. All the 12 individuals lie in the southern part of Similipal Tiger Reserve as indicated on the map.

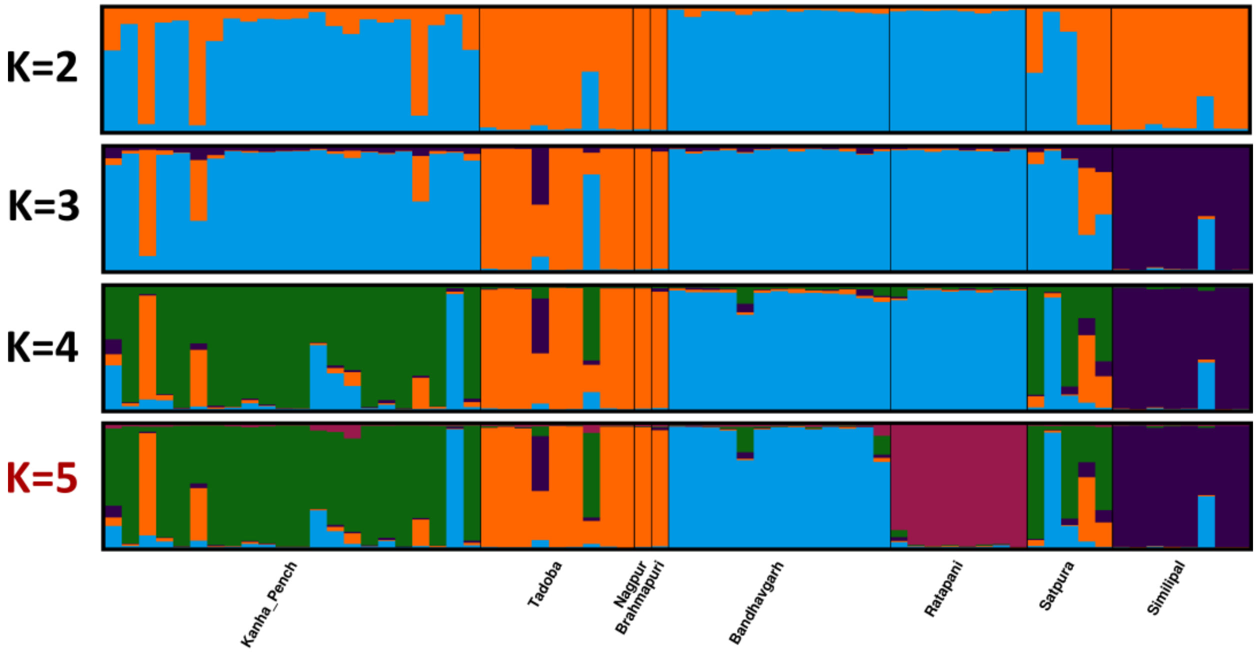

Fig. S4 – Sturucture analysis of Similipal tigers using data on 81 Loci along with central Indian tigers. Similipal separates out from other central Indian populations at K=3. The best K value = 5 is shown in red.

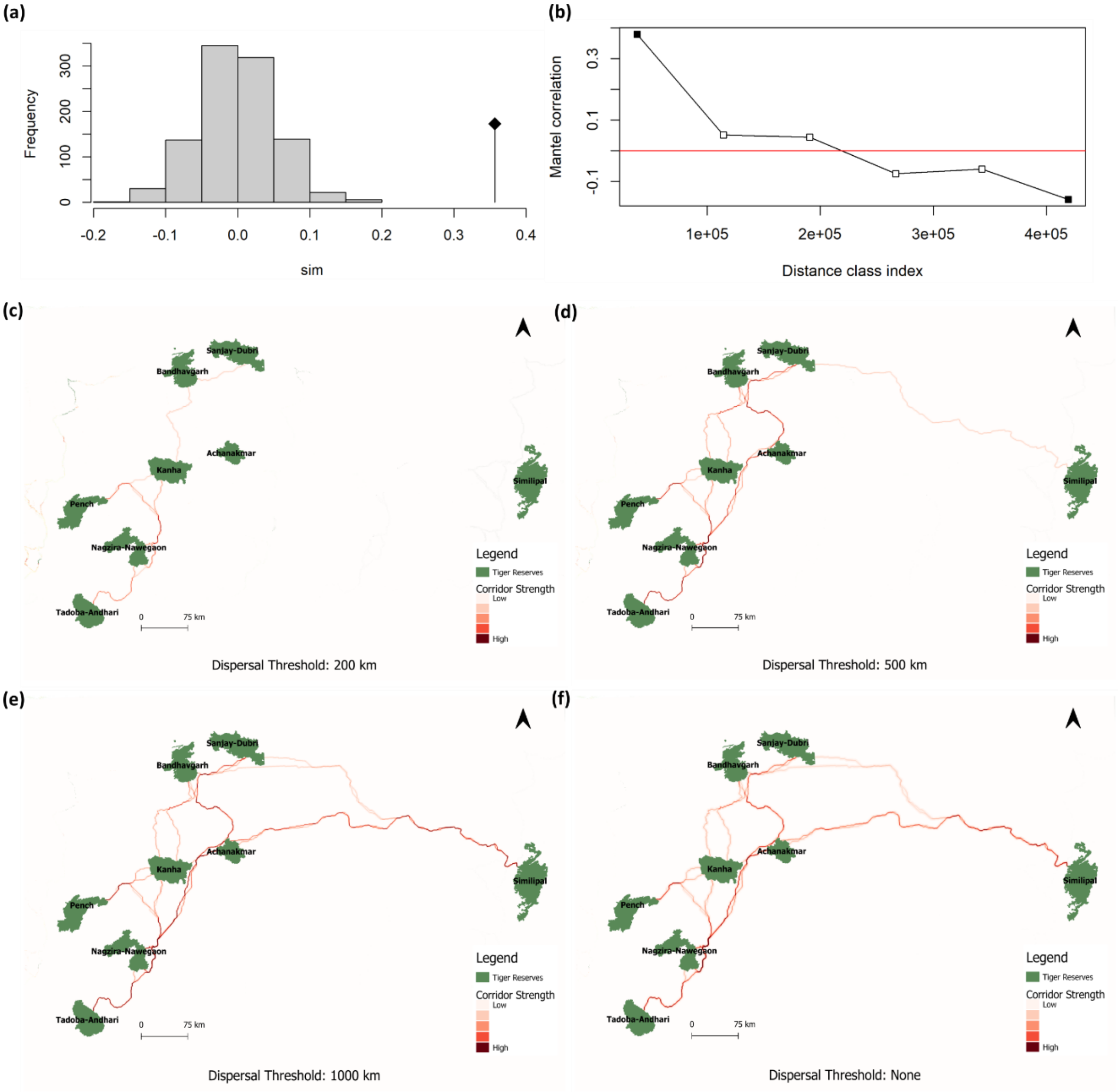

Fig. S5 – Similipal has reduced connectivity with other source populations. (a) Isolation by distance plot for central Indian tigers including Similipal. There is signification correlation between genetic distance and geographic distance between a pair of individuals (b) Mantel correlogram for geographic distance and genetic distance between a pair of individuals within central India. The correlation drops with increasing distance and becomes non-significant between 100 km and 350 km. (c, d, e, and f) Landscape analysis to estimate the resistance to connectivity using UNICOR factorial least cost paths based on the resistance surface with dispersal thresholds of 200 km, 500 km, and 1000 km. The output depicts location and strength of connections between nodes, in this case the tiger reserves. The strength of the connection between Simlipal and closest source populations is strong at 1000 km dispersal threshold, weak at 500 km and absent at the 200 km threshold.

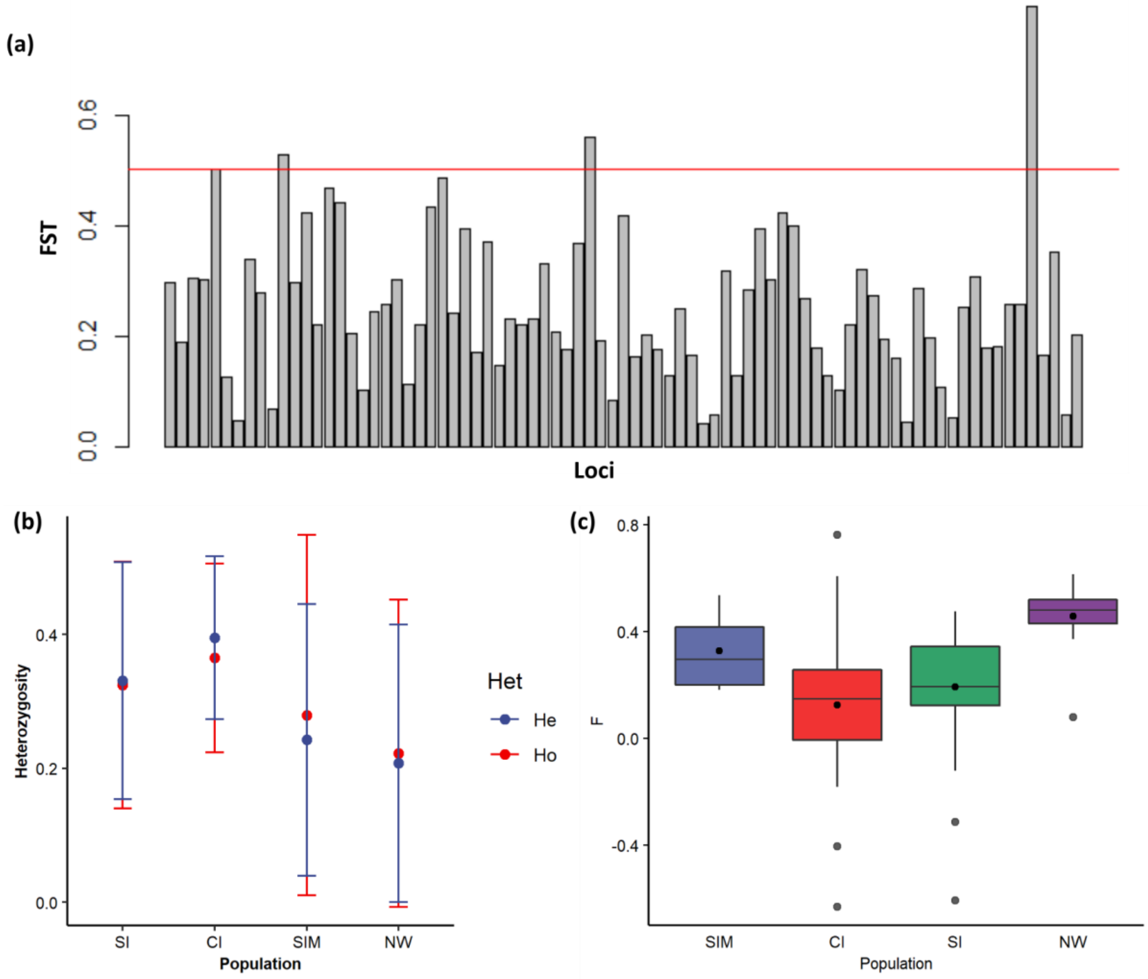

Fig. S6 – Population genetic analysis of Similipal tigers. (a) A distribution of pan India Weir and Cockerham's F_{ST} using data on 81 loci. The red line shows the F_{ST} value for Taqpep p. H454Y. Taqpep variant site appears in the top four loci but is not an outlier. (b) Distribution of observed and expected heterozygosity values at 81 neutral loci including *Taqpep* c.1360C>T mutation site across populations. Similipal (SIM) shows a low value of mean observed and expected heterozygosity similar to North-West India (NW), while Central India (CI) and South India (SI)show higher values of observed and expected heterozygosity (c) Population-wise distribution of the inbreeding coefficient of each individual estimated using genotypes at 81 neutral loci including *Taqpep* c.1360 site. Similipal (SIM, n=8) shows high positive values of F, similar to North-west India (NW, n=15) in contrast to Central India (CI, n=59), and South India (SI, n=42). The average value of F is indicated by the black dot (0.33 for Similipal, 0.46 for NW, 0.12 for CI and 0.19 for SI).

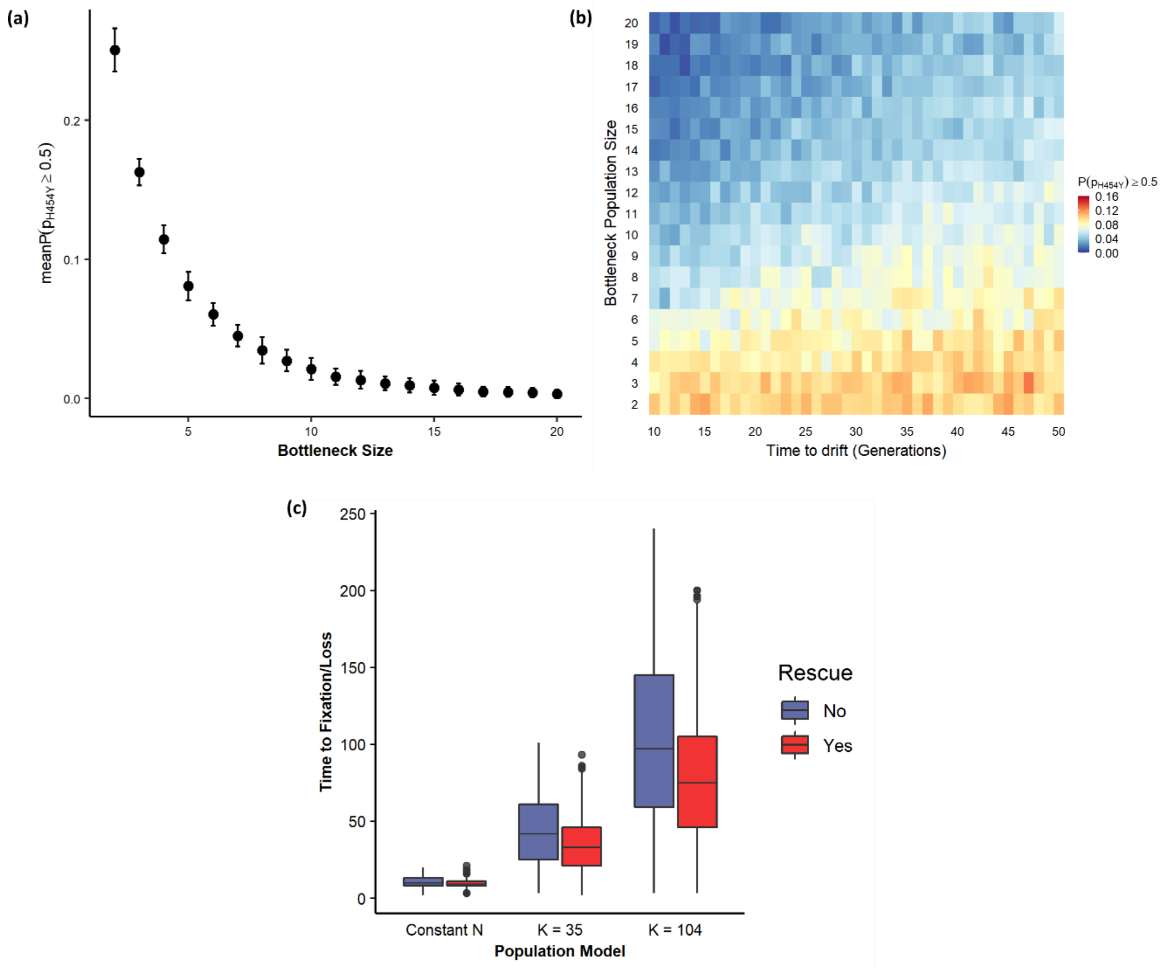

Fig. S7 – Population genetic analysis to understand the effect of drift and bottleneck population size on the frequency of the mutant allele (a) Distribution of bottleneck size vs the mean probability of mutant allele frequency >= 0.5 averaged over all values of allowed time to drift (10 – 50 generations). The error bars show the standard deviation. The error bars suggest that given a bottleneck size, time to drift does not affect the probability of mutant allele frequency reaching above 0.5 (c) Boxplot showing the time to fixation or loss of the mutant allele with starting frequency 0.5 in three population growth models, (i) No intrinsic growth (Constant N), (ii) Logistic growth with population carrying capacity 35 (K=35), and (iii) logistic growth with population carrying capacity 104 (K=104) under the effect of drift assuming isolation (No rescue) and genetic rescue with introduction of one wildtype homozygous individual every generation into the population.

Table S1. Reports of pseudomelanistic tigers in the past

S. No.	Year	Place	Description
1	1772	Kerala, India	Artist James Forbes depicted a black tiger killed in Kerala on a painting.
2	1846	Chittagong, Bangladesh	C. T. Buckland reported the killing of a melanistic tiger in Chittagong
3	1895	Travancore, India	Colonel S. Capper spotted a black tiger using a hunter's telescope
4	1914	Bhamo, Burma	A. T. Hauxwell shot at a black tiger, but it escaped and fled into the forest
5	1915	Assam, India	A black tiger was sighted east of Dibrugarh
6	1928	Assam, India	A dead black tiger reported from Lushai Hills
7	1936	Central Province, India	The skin of a tiger with thick black stripes and chocolate brown background was reported
8	1993	Odisha, India	A boy killed a black tigress in self-defense inside Similipal
9	2010	Chennai, India	A black tiger cub was born in Chennai Zoo
10	2014	Bhubaneswar, India	Two pseudomelanistic cubs were born to a white tigress and a normal orange tiger
11	2019	Chennai, India	Two pseudomelanistic cubs were born to white tigress and a normal orange tiger
12	2020	Ranchi, India	Two pseudomelanistic cubs were born

The table is compiled from the information given in Born Black: The melanistic tigers in India by L.A.K. Singh (1999)[1], and recent news on pseudomelanistic tigers

Table S2. Details of non-invasive samples collected from Similipal

S. No.	Species	No. of samples
1	*Panthera tigris*	62
2	*Panthera pardus*	43
3	*Felis chaus*	6
4	*Prionailurus bengalensis*	18
5	Undetermined	8
	**Total**	**137**

Table S3. Samples collected from Captive Tigers

**Sr. No.**	**Individual ID**	**Sample ID**					**Coat Type**	**Sex**	**Mutation Site Genotype****
		**Hair**	**Scat**	**Blood**	**Saliva**	**Tissue**			
Captive tigers from Nandankanan Zoological Park, Bhubaneswar, India									
1	NKB01	ZSH-01		**ZSB-01**		ZST-01	White Normal	Female	*+/+*
2	NKB02	ZSH-02	ZSSc-01		**ZSS-11**		White Normal	Female	*+/m*
3	NKB03		ZSSc-04		ZSS-08		White Normal	Female	*+/+*
4	NKB04	ZSH-07	ZSSc-05		ZSS-06		White Normal	Female	*+/m*
5	NKB05	ZSH-06	ZSSc-03		ZSS-07		Orange Normal	Female	*+/m*
6	NKB06		ZSSc-10		ZSS-04		White Normal	Male	*+/m*
7	NKB07	ZSH-04	ZSSc-08	**ZSB-05**			Orange Normal	Male	*+/m*
8	NKB08	ZSH-08	ZSSc-06		ZSS-03		Orange Normal	Female	*+/m*
9	NKB09	ZSH-11	ZSSc-12				Orange Normal	Female	*+/+*
10	NKB10		ZSSc-07		**ZSS-01**		Orange Pseudomelanistic	Male	*m/m*
11	NKB11	ZSH-10	ZSSc-11		**ZSS-02**		White Pseudomelanistic	Male	*m/m*
12	NKB12		ZSSc-13		ZSS-05		Orange Normal	Female	*+/m*
13	NKB13			**ZSB-04**			Orange Pseudomelanistic	Male	*m/m*
14	NKB14		ZSSc-02	**ZSB-02**			White Normal	Male	*+/+*
15	NKB15			**ZSB-03**			White Normal	Female	*+/m*
16	NKB16*					ZST-04	Orange Pseudomelanistic	Male	Undetermined
17	NKB17	ZSH-05	ZSSc-09		**ZSS-12**		Orange Normal	Male	*+/+*
Captive tigers from Arignar Anna Zoological Park, Chennai, India									
18	AAC01		CZSF-02				Orange Normal	Male	*+/m*
19	AAC02		CZSF-05				White Normal	Female	*+/m*
20	AAC03		CZSF-01				Orange Pseudomelanistic	Female	*m/m*
21	AAC04		CZSF-03				Orange Pseudomelanistic	Male	*m/m*
22	AAC05		CZSF-04				White Normal	Female	*+/m*

The samples used for whole genome sequencing are shown in bold face.
*A still-born cub was born after mating between NKB06 and NKB08, and was preserved in formalin at the zoo hospital. We collected the tissue sample from the formalin-preserved carcass of the individual. However, we were not able to get good quality DNA and genotype information from that because formalin affects the stability and integrity of the DNA.

**The symbol used for wildtype allele is '*+*' and that for mutant allele is '*m*'. Therefore, the three different genotypes obtained were *+/+*, *+/m*, and *m/m*. The pseudomelanistic tigers are homozygous for the mutant allele with the genotype *m/m*.

Table S4. Primers used in the study

S. No.	Primer Name	Primer Sequence
1	16S-rRNA-Cat F	AATTGACCTTCCCGTGAAGA
2	16S-rRNA-Cat R	TCCGACTGGTTAGTCTAGAT
3	Taqpep-c1360-SS161 F	TGGGGACTATTGATATTCGATG
4	Taqpep-c1360-SS161 R	AGCTGGAGAATATCTTCATGTG
5	Taqpep-c1360-ASPCR F	TGATGCAACCAAATGATCAAG
6	Taqpep-c1360-ASPCR-wt R	GCCTGATGTCCAATCTCTTG
7	Taqpep-c1360-ASPCR-mut R	GCCTGATGTCCAATCTCTTA
8	Taqpep-c1360-mPCR F	CGACAGGTTCAGAGTTCTACAGTCCGACGATCAGACAAAAAGGCTGTGATCTCC
9	Taqpep-c1360-mPCR R	GTGACTGGAGTTCAGACGTGTGCTCTTCCGATCTCACATGCCTGATGTCCAATC

Table S5: Output table of outlier test for selection

Locus	prob	log10(PO)	qval	alpha	fst
A1-14679996	0.122942	-0.853326	0.826743	0.011707	0.23168
A1-35119342	0.14384	-0.77467	0.80224	0.014979	0.2332
A1-54310529	0.1417	-0.78226	0.80375	-0.066175	0.22066
A1-55067172	0.147	-0.76362	0.79374	-0.06913	0.22067
A1-93741546	0.17084	-0.68604	0.75732	0.099678	0.24783
A1-94103495	0.1142	-0.88966	0.83289	-0.0044625	0.22885
A1-117967102	0.33321	-0.30128	0.57526	-0.41364	0.1865
A1-123674261	0.14892	-0.75701	0.79176	-0.071634	0.22012
A1-139418590	0.15954	-0.72164	0.77174	0.090588	0.24524
A1-193008632	0.14944	-0.75523	0.78972	-0.054316	0.22333
A1-229804014	0.11522	-0.8853	0.83119	0.027874	0.23384
A1-234082792	0.12368	-0.85035	0.82492	0.04291	0.23655
A1-235575823	0.1363	-0.80186	0.80933	-0.051525	0.22286
A2-25173709	0.11418	-0.88975	0.8337	-0.01133	0.22789
A2-101596561	0.10396	-0.93545	0.84359	0.005833	0.23012
A2-132629205	0.1183	-0.87233	0.82855	-0.024083	0.22601
A2-132896220	0.11088	-0.9041	0.83889	0.03222	0.23442
A2-141842481	0.19408	-0.6183	0.7099	-0.15234	0.2116
A3-17854948	0.14556	-0.76863	0.7974	0.061777	0.24006
A3-71227107	0.15008	-0.75305	0.78523	-0.07826	0.21918
A3-104768055	0.41473	-0.14959	0.52949	-0.57487	0.17093
A3-123388692	0.20742	-0.58218	0.66934	-0.16606	0.20978
B1-70034359	0.1328	-0.81491	0.81434	-0.045921	0.22344
B1-102119770	0.12148	-0.85924	0.82764	0.037314	0.23549
B1-112796118	0.10596	-0.92621	0.84292	0.011885	0.23102
B1-121664620	0.1454	-0.76919	0.79909	0.076143	0.24202
B1-124841777	0.10772	-0.9182	0.84226	0.016588	0.23189
B1-177950736	0.52629	0.045713	0.47371	-0.72202	0.15387
B1-180932493	0.16546	-0.70274	0.76139	0.075483	0.2438
B2-127265022	0.1267	-0.83838	0.82101	0.005664	0.23108
B2-136007523	0.10938	-0.91074	0.8416	0.0052769	0.23011
B3-5433236	0.17638	-0.66927	0.7531	0.11088	0.24905
B3-7385091	0.11346	-0.89285	0.83602	-0.010828	0.22787
B3-29347908	0.15004	-0.75318	0.78754	0.081345	0.24333
B3-43580915	0.11412	-0.89	0.83449	-0.010053	0.22806
B3-57525899	0.13604	-0.80282	0.81063	-0.050068	0.22307
B3-65026170	0.16454	-0.70564	0.76509	-0.095295	0.21716
B3-103738863	0.19296	-0.62142	0.72605	0.13833	0.25509
B3-128412472	0.11516	-0.88555	0.83205	0.039305	0.23558

B3-143092042	0.12906	-0.82919	0.8178	-0.020774	0.22712
B4-21318971	0.11152	-0.90129	0.83819	0.03534	0.23483
B4-112566143	0.11772	-0.87475	0.82945	0.028542	0.23405
C1-6854947	0.099462	-0.95684	0.84495	0.022459	0.23254
C1-8901194	0.11336	-0.89328	0.83675	0.018966	0.2324
C1-16726978	0.1817	-0.65355	0.74383	-0.12569	0.21423
C1-112973623	0.13098	-0.82181	0.81553	0.049697	0.23776
C1-151198145	0.12872	-0.8305	0.81889	-0.046075	0.22318
C1-182615900	0.14062	-0.78613	0.80658	-0.05616	0.22224
C1-191226737	0.16396	-0.70748	0.76847	-0.099914	0.21679
C1-200977673	0.11014	-0.90737	0.83959	7.71E-05	0.22935
C2-21916607	0.10944	-0.91048	0.84095	-0.013348	0.22728
C2-28023868	0.13806	-0.7954	0.80797	-0.039078	0.22492
C2-36803007	0.1552	-0.73585	0.78274	-0.075566	0.22041
C2-97858337	0.10986	-0.90861	0.84028	-0.0012627	0.2292
C2-135863436	0.12746	-0.8354	0.81997	-0.041047	0.22396
C2-138505992	0.30747	-0.35264	0.60458	0.30319	0.28511
C2-149157203	0.19024	-0.62904	0.73249	0.13456	0.25297
C2-154089352	0.15836	-0.72547	0.77478	-0.087958	0.21857
C2-156469471	0.29731	-0.37356	0.6242	-0.33332	0.19316
D1-1325306	0.15718	-0.72933	0.77761	-0.071612	0.22124
D1-19162879	0.19574	-0.61371	0.69923	-0.15009	0.21149
D1-37621837	0.11382	-0.89129	0.83526	-0.014449	0.22732
D1-38426736	0.12468	-0.84636	0.82397	-0.009918	0.22844
D1-65494160	0.13286	-0.81468	0.81314	0.056587	0.23894
D1-77531664	0.14146	-0.78312	0.80519	0.066616	0.24075
D1-94095398	0.22818	-0.52923	0.6488	-0.20005	0.20625
D2-11321268	0.1353	-0.80556	0.81188	0.059178	0.23942
D2-12871678	0.12634	-0.83979	0.823	0.050492	0.23761
D2-14681338	0.14454	-0.7722	0.8007	0.04886	0.23831
D3-14487396	0.19654	-0.6115	0.68611	-0.14791	0.21143
D3-56662506	0.1934	-0.62019	0.71869	-0.14089	0.21233
D4-44403987	0.17846	-0.66308	0.74869	0.12541	0.25172
D4-48407618	0.11758	-0.87533	0.83032	-0.032744	0.22459
E1-6409598	0.1308	-0.8225	0.81667	0.033159	0.23534
E1-17137334	0.10376	-0.93638	0.84425	0.0047061	0.22981
E1-53167804	0.12644	-0.8394	0.82202	0.031415	0.23493
E2-32058781	0.12318	-0.85236	0.82584	0.041897	0.23631
E2-34227145	0.1562	-0.73255	0.78026	-0.085972	0.21849
F1-31581208	0.14596	-0.76723	0.79562	0.075594	0.24223
F2-48430385	0.1832	-0.64918	0.73851	-0.11065	0.21668

F2-52805867	0.11294	-0.8951	0.83747	-0.004713	0.22867

Note - We used a Bayesian approach, implemented in Bayescan[2], to test for outlier loci on the entire dataset (81 loci, 124 individuals, described in Methods, section - Population genetics analyses - Wild tigers) initially with default run parameters and the prior odds for the neutral model set to 10. While higher values are recommended for larger data sets (1000s of SNPs), a prior odds of 10 seemed more suitable for our data set. However, even when we tested within central India samples and reduced the prior odds to 5, no outliers were detected (Results shown here). The lowest value of q-value detected in this run was 0.4737. So even at a FDR value of 40%, there are no outliers. *Taqpep* c.1360C>T is shown in red color.

Table – S6: Fisher's exact test p-values for Hardy Weinberg test at 81 loci

Locus	BTR	KPTR	SI	RTPN	RTR	SATP	SIM	TDB
A1-14679996	1	0.343	1	1	1	1	1	1
A1-35119342	1	1	1	1	1	1	1	1
A1-54310529	0.56	0.548	1	0.506	1	1	0.145	1
A1-55067172	0.575	1	0.231	0.499	1	1	1	0.428
A1-93741546	1	1	1	1	1	1	1	1
A1-94103495	1	0.372	0.516	1	0.513	1	1	0.086
A1-117967102	1	1	1	1	1	1	1	1
A1-123674261	0.284	1	0.187	1	0.073	1	1	1
A1-139418590	1	1	1	1	1	1	1	1
A1-193008632	1	1	0.308	1	1	1	1	1
A1-229804014	0.443	0.196	1	1	1	1	1	1
A1-234082792	1	1	0.029	1	1	1	1	1
A1-235575823	1	1	0.37	1	1	1	1	0.415
A2-25173709	0.523	0.376	0.015	1	1	1	0.468	1
A2-101596561	1	0.605	0.49	1	1	0.307	1	0.226
A2-132629205	1	0.128	1	1	1	1	1	1
A2-132896220	1	1	0.761	0.193	1	1	1	1
A2-141842481	0.146	0.62	0.663	1	1	1	1	1
A3-17854948	0.499	1	1	1	0.282	0.113	1	1
A3-71227107	1	0.383	0.316	0.484	1	0.436	1	1
A3-104768055	0.269	0.079	1	1	1	1	1	0.598
A3-123388692	0.021	1	1	0.203	1	0.119	0.07	1
B1-70034359	0.53	0.614	1	1	1	1	0.131	1
B1-102119770	1	0.134	1	1	0.583	1	1	0.488
B1-112796118	1	1	1	1	1	1	1	1
B1-121664620	1	0.2	0.231	1	1	0.431	1	1
B1-124841777	1	0.633	1	1	1	1	0.476	1
B1-177950736	0.564	0.613	1	1	1	0.327	1	0.268
B1-180932493	1	1	1	1	0.614	1	1	1
B2-127265022	1	1	0.74	1	1	1	1	1
B2-136007523	0.225	0.349	0.556	1	1	1	1	0.214
B3-5433236	1	0.164	0.402	1	1	1	1	1
B3-7385091	0.601	0.653	0.474	1	1	1	0.496	0.548
B3-29347908	1	0.164	1	1	1	1	1	0.205
B3-43580915	1	1	0.391	0.517	1	1	1	1
B3-57525899	1	1	1	0.471	1	1	1	1
B3-65026170	1	0.004	1	0.488	1	0.093	1	0.237
B3-103738863	1	1	1	1	1	1	1	1
B3-128412472	1	0.176	0.709	1	1	0.417	1	0.286

B3-143092042	0.224	0.345	0.328	1	1	1	1	1
B4-21318971	0.503	0.65	0.312	1	0.241	0.098	1	1
B4-112566143	1	1	0.662	1	0.214	1	1	0.268
C1-6854947	1	1	0.526	0.529	1	0.418	1	0.459
C1-8901194	1	1	0.375	1	1	1	0.44	1
C1-16726978	1	0.355	0.541	1	1	1	1	1
C1-112973623	1	1	0.181	1	0.619	0.107	1	0.56
C1-151198145	1	0.12	0.107	1	1	1	1	0.06
C1-182615900	1	0.627	0.321	1	1	0.445	1	0.526
C1-191226737	0.584	1	0.329	1	1	1	1	0.15
C1-200977673	0.289	0.124	0.512	1	1	1	0.483	1
C2-21916607	0.589	0.31	0.183	1	0.33	1	1	1
C2-28023868	NA	0.623	0.29	1	1	NA	1	1
C2-36803007	1	1	0.504	1	1	1	1	1
C2-97858337	0.1	1	0.065	1	1	1	1	1
C2-135863436	0.044	0.656	0.586	1	1	1	1	1
C2-138505992	1	1	1	1	1	1	1	1
C2-149157203	1	0.549	1	1	0.109	1	1	0.267
C2-154089352	0.239	0.648	0.751	1	1	1	1	0.535
C2-156469471	1	0.601	1	1	1	1	1	1
D1-1325306	1	1	1	1	1	1	1	1
D1-19162879	0.197	0.653	0.511	0.302	1	1	0.494	0.213
D1-37621837	1	1	0.745	1	1	1	1	1
D1-38426736	1	1	0.009	1	1	1	1	1
D1-65494160	1	1	0.076	1	1	1	1	1
D1-77531664	0.495	0.368	0.736	1	0.368	1	1	0.529
D1-94095398	0.165	0.608	0.76	0.436	1	1	0.432	0.549
D2-11321268	1	0.356	0.732	1	1	1	1	1
D2-12871678	1	0.682	0.761	1	1	0.318	1	0.281
D2-14681338	1	0.144	1	1	1	1	1	1
D3-14487396	1	0.673	0.129	0.445	0.306	1	0.077	0.525
D3-56662506	1	0.218	1	1	1	1	1	1
D4-44403987	1	1	1	0.202	0.634	1	1	0.53
D4-48407618	1	1	0.748	1	1	1	0.501	1
E1-6409598	1	1	0.282	1	1	1	1	1
E1-17137334	1	0.612	0.723	1	1	0.424	1	0.528
E1-53167804	1	0.518	1	1	1	1	1	0.194
E2-32058781	1	1	1	1	1	1	0.211	1
E2-34227145	1	0.54	0.233	1	1	0.352	0.059	1
F1-31581208	1	1	1	0.399	0.112	1	1	1
F2-48430385	1	1	1	1	1	1	1	0.147

F2-52805867	0.545	0.149	1	1	1	1	0.069	0.561

Note – A Fisher's exact test was done for Hardy Weinberg equilibrium for each protected area using *pegas* package in R. *Taqpep* c.1360C>T locus is shown in red color.

Abbreviations: BTR – Bandhavgarh, KPTR – Kanha-Pench, SI – South India, RTPN – Ratapani, RTR – Ranthambore, SATP – Satpura, SIM – Similipal, and TDB – Tadoba

SI References

1. Singh, L. A. K. *Born Black: The melanistic tigers in India*. (WWF-India, 1999).
2. Foll, M. & Gaggiotti, O. A Genome-Scan Method to Identify Selected Loci Appropriate for Both Dominant and Codominant Markers: A Bayesian Perspective. *Genetics* **180**, 977 LP – 993 (2008).
